# Reducing ocular *Demodex* using petroleum jelly may alleviate dry eye syndrome, blepharitis, facial dermatoses, ocular and respiratory allergies, and decrease associated prescribing: a hypothesis

**DOI:** 10.3389/falgy.2025.1576102

**Published:** 2025-08-20

**Authors:** Diana E. Senior-Fletcher

**Affiliations:** ^1^ Independent Research Pharmacist, Cardiff, United Kingdom; ^2^ The Demodex Project®, Cardiff Medicentre, Cardiff, United Kingdom

**Keywords:** *Demodex*, blepharitis, asthma, rhinitis, allergy, dermatitis, prescribing, petroleum jelly

## Abstract

*Demodex* eyelash mites are increasingly associated with eye and skin inflammation in humans, and cause demodectic mange in mammals. Informal accounts of symptom improvement and reduced need for anti-allergy medicines, when *Demodex* reproduction is prevented, indicate a further role linking *Demodex* to rhinitis, asthma and dermatitis. Their mobility, allergenic debris and consequential immunological impact may also explain progression of allergies in the *“allergic march”*. Being photophobic and nocturnal, *Demodex folliculorum* shelter, feed, and sleep in eyelash follicles during daylight. Coston (1967) speculated that *Demodex* emerge to mate during darkness and observed that medicated ointments rubbed into the eyelid margins at bedtime treated *Demodex* blepharitis effectively, presumably by preventing mating. Sixteen cases are described retrospectively whereby interested volunteers adopted Coston's technique, using unmedicated petroleum jelly. To break the lifecycle, a minimum 28-day course was advised, though concordance varied. Fourteen people reported relief from a surprising range of symptoms including not only dry eye and blepharitis but also rhinitis, asthma, angioedema and seborrhoeic dermatitis. Analysis of GP prescribing data in three volunteers allowed comparison of five-year periods immediately before and after starting continuous treatment. Mean yearly issues of anti-allergy and antimicrobial medicines reduced from 15.6 (range 8–25) to 1.8 (range 0–4), representing an 88.5% decrease for Volunteer 1 and from 5.8 (range 3–9) and 14.2 issues (range 9–24) to zero for both Volunteer 2 and Volunteer 13 respectively, representing 100% reductions in prescribing. Exacerbations of acne and dermatitis in two cases illustrate possible *Demodex* involvement in common dermatoses. This account is limited by its informal and retrospective nature in a disparate cohort, without assessment of *Demodex* levels. These preliminary observations support the hypothesis that *Demodex* allergens may trigger facial, ocular and respiratory inflammation and that reducing mite count with petroleum jelly improves symptoms. Formal clinical trials are needed to test this hypothesis.

## 1 Introduction – *Demodex* features, role in eye and skin disease, testing and treatment

**Figure 1:**
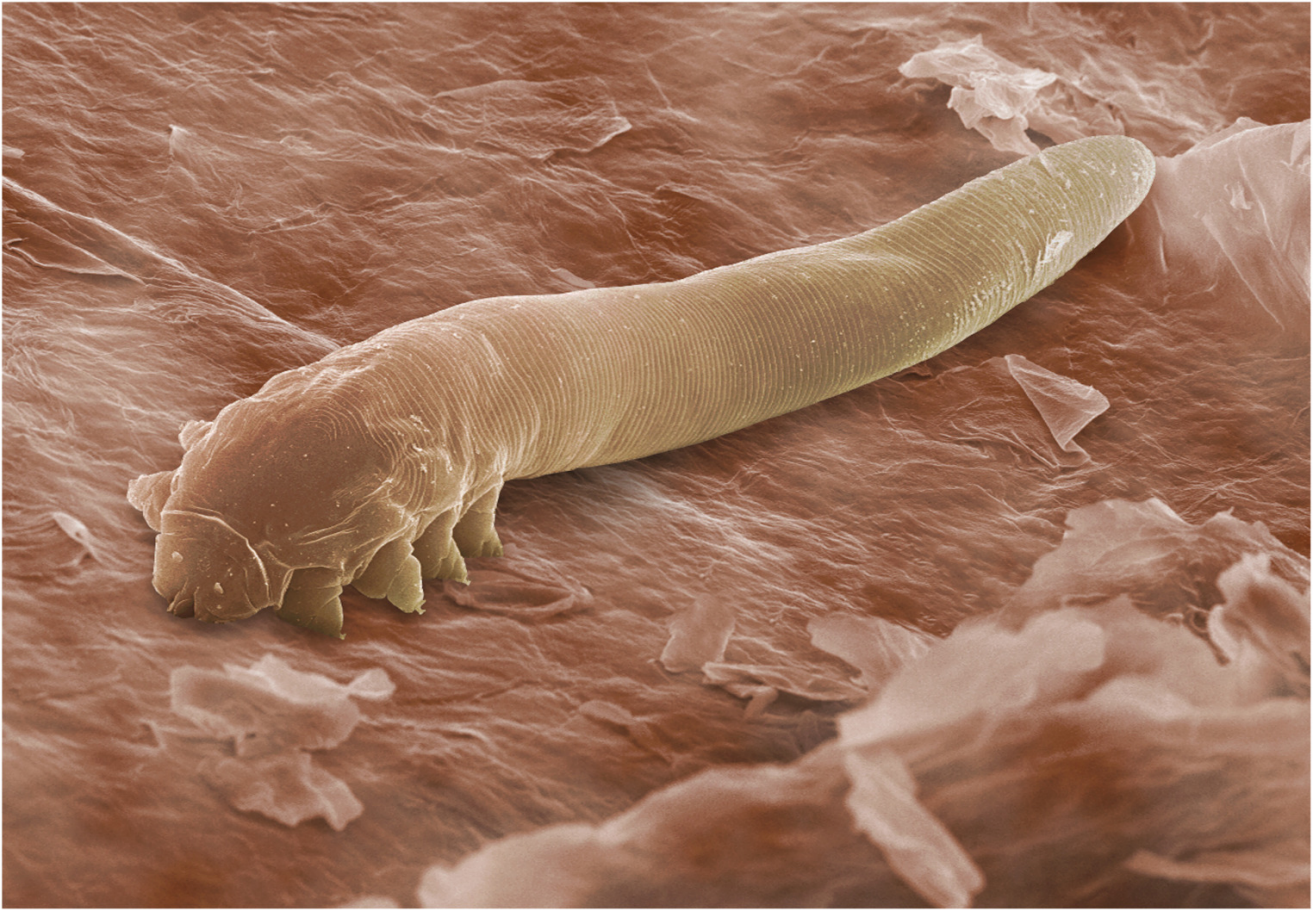
Scanning electron micrograph of an adult *Demodex folliculorum*. ©Power & Syred, Science Photo Library.

Classed as arachnids, *Demodex* (Figure 1) are eight-legged arthropods closely related to spiders and scorpions. Their subclass, the “*Acari*” includes ticks and other mites, including house dust mites (HDMs), with their well-recognised allergenic potential, “chiggers”, or “harvest mites”, and scabies (1) all of which impact human health (2). *Demodex* are stated to be the most common parasite in human skin and eyes (3), generally assumed to be referring to the eye adnexa. They are also the most highly evolved microorganisms within the human microbiome (4), possibly pre-dating or evolving alongside early humans (5). Transmission is predominantly vertical through close maternal contact (6, 7).

**Figure 2:**
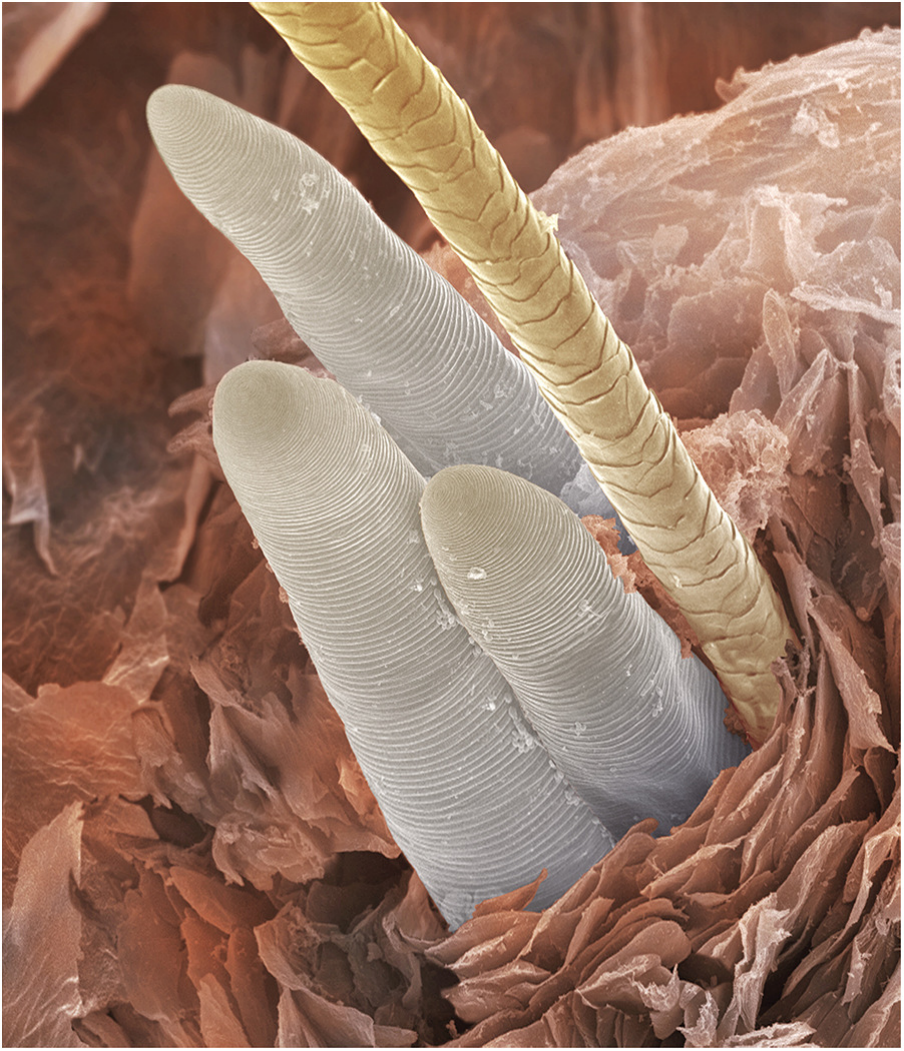
Scanning electron micrograph showing *Demodex* tails protruding from a hair follicle. ©Power & Syred, Science Photo Library.

Two closely related species are found in humans: worm-shaped *Demodex folliculorum* which group together in eyelash and hair follicles (Figure 2) and their shorter, solitary cousins, *Demodex brevis**^1^***. The latter live deep in dermal sebaceous glands, and in the lipid-secreting meibomian glands on the inner eyelid margin (3).

Many detailed reviews on the features, behaviour and means of eradicating *Demodex* mites have been published over the years, (3, 8–24). This article describes the effects of using a technique thought to reduce ocular *Demodex* and how this has led to a hypothesis which proposes a causative role for *Demodex* in respiratory and dermatological inflammation.

*Demodex* are known to cause demodectic mange in dogs and other mammals (25, 26) and are associated with chronic inflammatory skin conditions in humans (4), including rosacea (27–29), acne (29–31), seborrhoeic dermatitis (29) pityriasis, peri-oral dermatitis, facial pigmentation and scalp eruptions (3). “Primary demodicosis” usually affects the elderly, typically appearing round the mouth, whereas the more diffuse 'secondary' form affects the face and trunk, often in younger people (4). *Demodex* infestation increases with age, from 84% of the population at age 60, to 100% in people over 70 years (32). They proliferate when immunity is compromised (4, 18), during puberty, and at other times of increased production of sebum (26), which is a key nutrient (6). This possibly explains exacerbation of *Demodex*-related skin conditions in adolescents and at times of stress.

*Demodex* have highly developed mechanisms for evading immune attack (33–35), but they also evade suspicion in clinical and research settings, being found on healthy people and animals as well as those suffering significant illness. Direct causation of illness is consequently difficult to establish (26). *Demodex* also have many potential mechanisms for causing tissue damage and immunological reactions (3, 14, 26), such as release of digestive enzymes^2^ and allergenic *Der f* proteins (36) and their role as vectors for other microbes^3^.

In eye disease, *Demodex* infestation is associated with blepharitis (18, 37, 38), dry eye syndrome^4^ (18, 39, 40), meibomian gland disease (MGD) (3, 41) keratoconjunctivitis sicca (27, 42–44), conjunctivitis, keratitis and pterygium, or “Surfer's Eye” (3). They are also found in higher density in patients with basal cell carcinoma of the eyelid (45).

Blepharitis is a common inflammatory eye condition across all ages and all ethnic groups (46), predominantly developing in middle age but also affecting children. It occurs in 37% of ophthalmology cases and 47% of optometrist cases and is classed as either “anterior”, for eyelash follicle involvement, “posterior” for meibomian gland blockade, or “mixed”^5^. Symptoms include itching, redness, flaking, and crusting of the eyelids. Progression may involve damage to the eyelid margins and cornea through increased vascularity and ulceration leading to photophobia, blurred vision and sight loss. Burning sensations and irritation are significant problems, often accompanied by increased lachrymation and erythema (4).

While a connection is not always considered, accepted signs of *Demodex* infestation mirror symptoms of blepharitis, namely cylindrical dandruff, scaly or waxy debris on the eyelashes, eyelid erythema and increased eyelid vascularity (42). However, lash epilation has also revealed *Demodex* infestation in a blepharitis patient with unilateral fine follicular scaling (47) and in blepharo-keratoconjunctivitis patients with clean lashes (42). Distension or “pouting” of eyelash follicles has also been reported as a pathognomonic sign (22); eyelash loss (madarosis) and inward turning of the lashes (trichiasis) causing corneal abrasion may also be seen (3). Individuals with *Demodex* are more likely to report itching (39, 48) and ocular discomfort, which correlates closely with infestation levels (48, 49). While lash epilation for microscopy has been used in many studies, eyelash rotation (50) and the “lateral eyelash tension technique” (51) have been found to achieve higher counts, allowing mites to be counted *in situ,* without removal of lashes. These techniques require only a slit lamp, good tweezers, and a steady hand.

Various treatments have been suggested for eradicating ocular *Demodex* and treating blepharitis though no formal consensus on the best option has been reached (52). The NICE Clinical Knowledge Summary for blepharitis^5^ does not include *Demodex* as a potential cause but recommends eyelid hygiene, warm compresses and eyelid massage as symptomatic treatments. Oral and topical antimicrobials are allowed where necessary. NICE also advises that a cure is generally not possible, though evidence for the efficacy of a 3-month course of topical ivermectin 1.0% cream in treating ocular demodicosis has been shown (22). Lotilaner is now available as eye drops for *Demodex* blepharitis. Previously it was exclusively a veterinary medicine for killing ticks, mites and fleas at any stage of the lifecycle (53).

In 1967 Tullos D. Coston published a detailed review of the lifecycle and behaviour of *Demodex* mites and appraised potential treatments for *Demodex* blepharitis (10). Many would be considered noxious by today's standards, but he noticed that ointments spread in the eyelashes overnight were particularly effective. He also suspected that *Demodex* emerge from eyelash follicles during darkness to copulate. Our first volunteer had suffered for more than thirty years with debilitating dry eye disease and blepharitis^6^. She agreed to try using Vaseline®, applied in this way at night, to physically prevent mite reproduction. The surprising results are summarised below and described fully in Supplementary S2.

It was later discovered that the same technique is also used by a very small number of clinicians, in conjunction with tea tree oil wipes, heated eye masks, or as monotherapy for blepharitis, MGD and dry eye symptoms. A GP has reported success with the technique for *Demodex* blepharitis (54), and LJ Geisse has described using it for “hundreds of patients” over a three-year period^7^, although not in the context of reducing *Demodex*. There appear to be no other reports of Vaseline®, known generically as “petroleum jelly”, “white soft paraffin” or “petrolatum”, being used in this very specific way at bedtime. It is used for *Phthirus pubis*, or “crab lice”, applied four times a day when ocular infestation occurs (55).

A link between *Demodex* proteins, rhinitis and sinusitis has already been proposed^8^ and high levels of gravid *Demodex* have been reported in nasal discharge (56). Both support the likelihood that *Demodex* and their associated proteins will infiltrate the respiratory and gastrointestinal systems.

## 2 Methods – collection of cases and application of petroleum jelly to trap *Demodex*

Upon learning of this topic, personal contacts of the author often express a wish to try the treatment for themselves. Between 2013 and 2023, sixteen individuals, many being healthcare professionals, volunteered to try Coston's technique. Initially, their use was for dry eye symptoms or blepharitis but later progressed, as experience increased, to include allergic eye conditions, rhinitis, and asthma. All were given verbal advice to follow a simple routine immediately before going to sleep in a dark room. Instructions comprised careful handwashing then taking a large pea-sized amount of petroleum jelly with a fingertip and gently working it into the base of their eyelashes along the margins of their firmly closed eyelids. The aim of creating a glutinous environment within the eyelashes to trap mites as they emerge at night to mate was explained. Applied in this way, the petroleum jelly does not get inside the lids or contact the ocular surface and may be removed the following morning. Advice to use the treatment for at least 28 consecutive nights was given, to exceed the *Demodex* lifecycle of 14–21 days (9, 57). Except for Volunteer 15, everyone was aware of the treatment rationale. Retrospectively, every volunteer, or their legal guardian, emailed consent for their case reports and photographs to be published.

## 3 Case reports – volunteers with dry eye syndrome, blepharitis, rhinitis, allergies, acne & asthma

Please see Table 1 for a summary of medical history, use of the Coston Treatment and outcomes. For full description and clinical images, please see Supplementary S2.

**Table 1:** Summary of medical history, use of the Coston treatment and outcomes.

Volunteer demographics	Current problems & medical history (PMH), where available	Medication history (where available, and not complete)	Coston treatment regimen.	Results—short and long term
V1 Female aged 84. Please see images: Supplementary S2 Figures 1A,B See graph of prescriptions issued Figure 4A	Severe dry eye & blepharitis, severe periorificial seborrhoeic dermatitis (eyes, ears & mouth), atopic dermatitis on back, migraines, arthritis & osteoporosis, hypertension, mixed Alzheimer's & vascular dementia, aortic stenosis, early cataracts. PMH Thyroidectomy, gastric ulcer perforation, truncal vagotomy & pyloroplasty, adverse reactions (ADRs) to beta blockers (dry eye), statins (muscle pain).	**Regular for:** Gastritis, hypertension, osteoporosis, thyroid hormone replacement. **As requested:** Ocular antibiotics (x6 in 2012), ocular lubricants (x12 in 2012), tacrolimus ointment, topical and oral antibiotics (multiple courses for eye & skin infections), antihistamines, topical antifungals and steroids for atopic/infected dermatitis.	Started late 2013, nightly, supervised. After first month, reduced to alternate or 1 in 3 nights till end of life. (V1 died in November 2019, aged 91, of pneumonia post hip fracture & intra-operative stroke.)	**Short term** Blepharitis and seborrhoeic dermatitis Improved dramatically within a few days. No further prescription of ocular lubricants, topical or oral antibiotics for eye or skin conditions, or antihistamines. **Long term** Cataracts, diagnosed in 2012, did not progress and vision remained clear. General health improved, allowing the proton pump inhibitors for her gastritis to be stopped, migraines resolved, arthritis in her hands improved, dementia did not deteriorate beyond short term memory loss.
V2 Schoolboy, aged 13. See graph of prescriptions issued Figure 4B	Blepharitis (itching, inflammation & lash debris) (4-year history), rhinitis & hypertrophy of nasal mucosa (surgery offered), seborrhoeic dermatitis, asthma, allergy to cats, grasses, and house dust mites.	Corticosteroid nasal sprays, oral antihistamines, inhaled corticosteroids and salbutamol, topical antimicrobials.	Started 2014 at the age of 13. Continued every night into adulthood, reporting that it prevents return of his allergy symptoms.	**Short term** Eye symptoms resolved, nasal obstruction & rhinitis cleared fully within 3–4 days. **Long term** Noticed asthma had resolved. One issue of antihistamines and one of salbutamol in 2014 were precautionary and not used.
V3 Male healthcare worker (HCW), aged 58.	Facial acne, seborrheic dermatitis, chronic rhinitis since age 14.	Not disclosed.	Started in 2017, retreated when symptoms returned initially but lapsed long term. In 2024 he reported mixed psoriasis and seborrhoeic dermatitis, treated with topical tacrolimus.	**Short term** Facial acne and seborrheic dermatitis resolved quickly, nasal congestion cleared shortly afterwards. **Not used long term**
V4 Male HCW, aged 50.	Seborrhoeic dermatitis, nasal congestion.	Not disclosed.	Commenced in 2018. In 2024 he reported not needing to use the treatment but was prepared to do so if symptoms returned.	**Short term** Nasal congestion resolved, seborrhoeic dermatitis resolved.
V5 Female student, aged 16. Please see images: Supplementary S2 Figures 2A,B	Moderate chronic facial acne, dry eye syndrome.	Not disclosed.	Continuous long-term use on lashes and directly on acne on face and upper body.	**Short term** Rapid resolution in acne, optometrist coincidentally reported resolution of dry eye syndrome. **Long Term (after 2 years continuous use)** Outbreak of acne on her back. [Indicating that *Demodex* may migrate to survive (58).]
V6 Female HCW, aged 23.	Idiopathic angioedema, eczema, hay fever.	Emergency hospital treatment with corticosteroids & high dose fexofenadine.	Full 28-day course (2017) then stopped. No recurrence, no further antihistamines required. (Confirmed 2020.)	**Short term & long term** Angioedema resolved, allowing withdrawal of antihistamine tablets over several days.
V7 Female HCW, aged 23. Please see images: Supplementary S2 Figures 3A-E	Severe itching and erythema around eyes, ears, and mouth with a well-defined map-like edge. No specific allergens identified. PMH Mild eczema since childhood, four allergic reactions affecting the face and eyes, since the age of 19, severe conjunctivitis.	Potent topical steroids, antifungal creams, tacrolimus ointment (all ineffective), oral prednisolone (effective), Conjunctivitis: Topical antibiotics & steroids (ineffective), sodium cromoglicate (effective).	Started treatment during a symptom flare. Two years later, another flare around the eyes required treatment with oral steroids. Subsequently, she re-commenced the Coston treatment and continues to use it intermittently whenever a flare is developing.	**Short term** Without further application, she remained symptom-free for two years. **Long term** She reports that it has reduced the severity of her flares which usually subside within a few days of restarting the treatment. Eczema on her arms has also resolved after using heavy ointments at night, in a similar approach to the ocular treatment.
V8 Schoolgirl, aged 9.	Severe blepharitis with visible lash dandruff, frequent styes, asthma-like nocturnal cough requiring multiple GP visits, exercise-induced asthma, allergy to horses.	Eyelid hygiene and oral antihistamines (ineffective), thermal eye mask, during school breaks, inhaled steroids and salbutamol. [Refused topical steroids & oral antibiotics]	Treatment was supervised by mother, who noticed that blepharitis flared whenever treatment lapsed, and that it resolved within three days of restarting supervision.	**Short term (one week)** Dramatic improvement in blepharitis linked with compliance. **Long term (three months)** Nocturnal cough resolved, no further styes, improved sleep quality, growth in stature of ’several inches’ over 4–6 months.
V9 Schoolboy, aged 11, brother of V8.	Two-year history of: asthma, mild blepharitis, styes with pain and itching, nasal congestion & snoring, itching round his ears, which were “frequently blocked”.	Inhaled steroids, salbutamol.	Treatment was again supervised by mother. As with V8, she noticed symptoms returned when she didn’t monitor application, and that control returned within 3 days of re-starting supervision.	**Short term** Improvement ‘within a few days’ in blepharitis, nasal congestion and peri-auricular itching. **Long term** Asthma medication was no longer required, no further styes, snoring resolved during treatment, rapid growth in stature.
V10 Female non-HCW, late 40s, parent of V8 & V9.	Eye inflammation on waking, full body itching & ‘hives’, earache, poor sleep & night sweats, anxiety, boils, recent hospitalisation for COVID-19. PMH Asthma from age 18, severe allergy to horses	Inhaled steroids and beta 2 agonists for asthma, other items not disclosed.	Continuous long-term use.	**Short term (8–9 days**) Eye, skin and chest symptoms cleared, improved sleep. **Longer term (3 weeks)** Asthma resolved with no further need for ‘reliever’ medication, skin symptoms improved - no further nocturnal itching. **Long term (18 months)** No further need for inhaled steroids, no further eye inflammation.
V11 Female HCW & author, aged 41.	Two-year history of: Multiple allergies, severe dry eye syndrome preventing contact lens use and making screen work very uncomfortable, significant eye inflammation on waking, frequent episodes of conjunctivitis and recurrent eye infections.	Antihistamines, ocular lubricants, sodium cromoglicate, increased fluid intake (all without benefit).	Started treatment in 2014, repeated ‘whenever symptoms returned’.	**Short term (1 day to 1 week)** Relief of ocular discomfort and inflammation (after 1st use), dry eye symptoms resolved fully, allowing return to contact lens use. **Long term (follow up 2024** No further medication required for dry eye syndrome, allergies, hay fever or eye infections, comfortable use of contact lenses.
V12 HCW, aged 52	Long term dry eye syndrome, uveitis, dendritic corneal ulcers at times of stress, rheumatoid arthritis (RA), ’sight-threatening viral keratitis’. PMH Anterior uveitis age 6. (Parents were warned of RA likelihood in later life.)	Steroid, antibiotic and antiviral eye drops (long term), methotrexate.	Started in 2017, used continuously long term, every night.	**Long term** Finds it prevents dry eyes and ocular irritation, ophthalmologist has noted resolution of keratitis and stated that her eyesight is no longer in danger, reduced joint stiffness and pain related to her RA noticed in relation to Coston treatment.
V13 Female student, aged 18.	Asthma-like symptoms since infancy. Rhinitis, acne, gastritis, and headaches from the age of 10, apparently coincident with developing acne on her forehead.	Antihistamines—eye drops & oral, asthma inhalers, topical antimicrobials, emollients, steroid nasal sprays, acne treatments, topical. **Asthma flare while abroad 2016–7** Inhaled high dose steroids, long-acting beta 2 agonists and leukotriene agonist. Oral prednisolone for severe asthma.	Started 2015 Treatment lapsed during her second year 2016–7, while studying abroad. Restarted Coston treatment on return to the UK on an unrecorded date in late 2018 or early 2019	**Short term** Successfully resolved her rhinitis and asthma, prior to starting university While abroad and sharing accommodation with cigarette smokers her asthma flared, requiring emergency courses of oral prednisolone, and return to full treatment. **Coston Retreatment from 2018/19** allowed withdrawal of her asthma and rhinitis medications slowly and completely over a period of a few weeks without further relapse. **Note:** While prescribing data are incomplete due to living abroad again from 2019 to 22, she confirmed she had no further need for asthma treatments during that period.
V14 Female non-HCW Age c40	Dry eye syndrome with itching, watery, visibly sore eyes aggravated by cosmetics, severe seasonal hay fever featuring sneezing and rhinorrhoea.	Not disclosed.	Started in 2022 and used continuously, long term, to prevent symptom return.	**Short term (few days)** Eye symptoms greatly improved with no further itching or inflammation. **Long term (2 years)** In 2024 she reported not experiencing any hay fever symptoms that year, to her great surprise.
V15 Female non-HCW Age c 28 Please see image: Supplementary S2 Figure 4	Severe acne since teenage.	Not disclosed.	Used short term but stopped when acne flared within a few days.	**Short term** **Severe flare of acne** featuring a deep crack at the corner of her mouth, recovered gradually without intervention after stopping Coston treatment.
V16 Male scientist, aged 60. Please see image: Supplementary S2 Figure 5	Dry, itching eyes, type 1 diabetes, chronic eczema of the face, scalp and body, hay fever.	Multiple medicines for diabetes, eczema and allergies. (Detail not disclosed.) Flare treated with oral prednisolone and topical steroids.	Initial period of application to eyelashes proved ineffective. Subsequently, he increased the area treated, to include the full face and scalp.	**Short term** Little initial effect from regular use of the Coston treatment. **Wider use triggered a severe full-body flare** with intense itching and raised excoriated patches, even on untreated areas. Note: At V16's request, a dermatologist performed a gentle, and therefore probably ineffective, skin scrape, but no *Demodex* were found.

For full description and clinical images please see Supplementary S2.

## 4 Results – resolution of symptoms and reduction in GP prescribing

Table 2 describes the conditions relieved or exacerbated in the volunteers following use of petroleum jelly to reduce ocular *Demodex*.

**Table 2:** Conditions relieved or exacerbated in the volunteers following use of petroleum jelly to reduce ocular *demodex*.

Condition (Total number affected)	Volunteers reporting relief.	Volunteers reporting either no benefit or an exacerbation.
Acne (3)	V3, V5	V5 (Initial improvement then migration.) V15 (Severe flare)
Angioedema (1)	V6	–
Asthma (5)	V2, V8, V9, V10, V13	–
Blepharitis (4)	V1, V2, V8, V9	–
Cataracts (1)	V1	–
Conjunctivitis (2)	V7, V11	–
Dry eye syndrome with itching (7)	V1, V2, V5, V11, V12, V14	V16 (Severe eczema/dermatitis following wider application.)
Ear itch, canal debris build-up (1)	V9	–
Earache (1)	V10	–
Erythema around eyes, nose, and mouth (1)	V7	–
Eyelid swelling & itching (2)	V10, V11	–
Hay fever (1)	V14	–
Keratitis (1)	V12	–
Nasal congestion/rhinitis (5)	V2, V3, V4, V9, V13	–
Poor sleep quality (3)	V8, V9, V10	–
Seborrhoeic dermatitis (3)	V1, V3, V4	–
Styes (2)	V8, V9	–

The self-administered Coston technique, using petroleum jelly, was initially proposed for dry eye symptoms or blepharitis. All cases of dry eye or blepharitis resolved except one, V16, who used the treatment more widely and suffered a severe full body flare of his pre-existing dermatitis. Four further volunteers with a history of severe allergic ocular symptoms, including one with recurrent angioedema (V6, V7, V10, V11), also reported full resolution, as did all five people who had suffered chronic nasal congestion (V2, V3, V4, V9 & V13). In the five volunteers who had asthma as a comorbidity to eye symptoms (V2, V8, V9, V10 & V13), the improvement was an unexpected observation, while V13 later restarted the petroleum jelly in a successful concerted attempt to resolve her asthma and stop her medication.

When the volunteers used the treatment they experienced resolution of blepharitis, dry eye, ocular allergies and infections, keratitis, hay fever and respiratory inflammation in the form of rhinitis and asthma. Other incidental findings included resolution of seborrhoeic dermatitis in three volunteers (V1, V3 & V4), relief of earache and ear itch in one volunteer each (V9, V10), and deeper sleep, reported in three members of the same family (V8, V9, V10). V14 reported absence of hay fever symptoms in the second year of treatment, V1's cataracts did not develop over the following seven years and V12's resolution of keratitis was also unexpected.

A reduced need for prescribed and purchased medicines was reported in at least 11 volunteers. This was verified in three volunteers by analysis of their GP prescribing data. For V1, ocular lubricants, topical immunosuppressants, topical and oral antibiotics, antihistamines and, subsequently, medication for gastritis were no longer requested. For V2 and V13, medications no longer needed included nasal steroids and asthma treatments.

The volunteers generally described their improvements as total; no one reported mild or moderate improvement for any condition. Volunteers found that symptoms correlated closely with use of the treatment, and that their symptoms returned if compliance was poor. V6, who achieved 28 nights of continuous treatment for her angioedema, was able to withdraw her high-dose antihistamines without relapse, and without needing further treatment.

These results are surprising, considering the simple physical technique involving an inert substance. While these cases do not provide direct evidence of reduction in *Demodex* levels, the reported correlation between treatment and symptom resolution indicates a probable association. Improvement in eye and skin conditions could be attributed to a lubricant, emollient, or placebo effect, or to spontaneous fluctuations. However, resolution of nasal congestion and asthma is harder to explain. It points strongly to these conditions being caused or exacerbated by inflammatory proteins, including some identified as *Der f* house dust mite-type allergens (36), leeching from debris left by ocular *Demodex*.

## 5 Hypothesis – *Demodex* allergens may cause respiratory inflammation

When *Demodex* mites disintegrate in the eye, inflammatory *Der f* proteins and other allergenic debris will drain into the nasal passages via lachrymal fluid and be inhaled. This may trigger inflammatory reactions including rhinitis and asthma. Established links between ocular and respiratory inflammation may therefore be explained.

Eradicating ocular *Demodex* with petroleum jelly, applied at night to impede mating, may provide a new approach to first-line management of dry eye, blepharitis, rhinitis, asthma and other allergic conditions, improving quality of life and reducing medication needs, prescribing costs and clinic time.

## 6 Discussion

An association between eye inflammation in the form of “allergic conjunctivitis” with rhinitis and asthma has been described by Sánchez-Hernández et al. (59). They found that allergic conjunctivitis occurred in 88.3% of patients with rhinitis and in 38.8% of patients with asthma. Paediatric results were higher at 93% and 47% respectively. Asthma and rhinitis correlated with conjunctivitis in severity and duration, demonstrating the “*allergic march”* and supporting the “one airway, one disease” hypothesis that asthma onset follows allergic conjunctivitis and rhinitis (59). Awareness that ocular demodicosis may be misdiagnosed as allergic conjunctivitis, viral keratitis or other inflammatory eye conditions (42) increases the relevance of this information and strengthens the case for a shared underlying aetiology between eye inflammation and allergic respiratory conditions.

The resolution in seborrhoeic dermatitis and changes in acne and atopic dermatitis were all unexpected, though all three conditions have already been associated with *Demodex* (29) and association between *Demodex* and these and other skin conditions including rosacea and pityriasis folliculorum, appears to be gaining recognition (29, 60).

Three volunteers (V1, V3, V4) who had coincidental seborrhoeic dermatitis used petroleum jelly only expecting to treat their eye or nasal symptoms. The jelly was applied only in the periocular region, yet facial seborrhoeic dermatitis resolved in all three volunteers, alongside some improvement in acne in V3. This suggests that the “*allergic march”* may start with ocular *Demodex*. Their mobility and 'site specificity', particularly in the sebaceous periorificial regions (4, 10), make it plausible that *Demodex* could migrate from the ocular area to trigger skin inflammation. Concomitantly their allergenic debris would pervade the respiratory tract as the *Demodex* population rises, offering a mechanism whereby dermatitis, rhinitis and asthma are related. As *Demodex* are transmitted primarily through close maternal contact (5), familial traits in atopic diseases may be explained. We should also consider that once allergenic proteins enter the nasal passages they will be swallowed, perhaps explaining the link between eye inflammation and inflammatory bowel diseases (61, 62), as noted by Burrill Crohn in 1925 (63) and possibly as seen in V1.

Formal measurement of *Demodex* levels and their debris in the skin and respiratory tract before and after the Coston treatment could strengthen the evidence for *Demodex* causing seborrhoeic dermatitis, acneiform conditions, atopic dermatitis and other associated dermatoses. It may also inform the current debate on where the “*allergic march”* originates (64). Whether treating these skin conditions conversely reduces ocular inflammation was not elucidated in these volunteers, but merits investigation.

### 6.1 Risk of exacerbation in acne and atopic dermatitis

Skin flares are well-known features of acne and atopic dermatitis, often in response to identifiable environmental changes, including temperature and psychological stress though the reactivity of ectoparasites is not yet commonly considered as a potential mechanism^9^. As petroleum jelly is inert, the eruptions of acne in V15 and dermatitis in V16, which developed in untreated areas, are more easily explained as a “threat response” rather than a pharmacological adverse reaction. It demonstrates that an environmental threat may cause *Demodex* to react *en masse* or to migrate to a safer location, and that a “hornets” nest approach” to treatment may be prudent. A role for *Demodex* in atopic dermatitis is now recognised (65, 66), and the clinical parallels between canine demodicosis and some human skin conditions are striking^10^.

### 6.2 *Demodex* and acne

**Figure 3:**
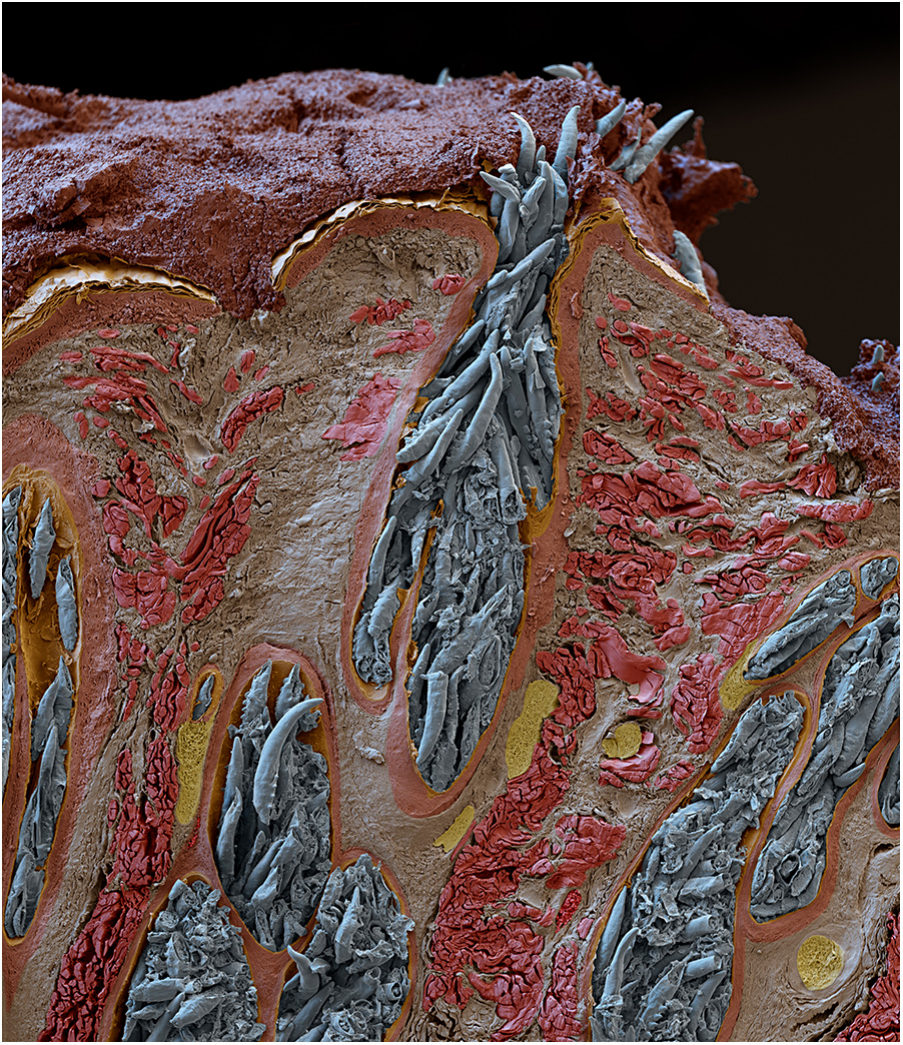
Scanning electron micrograph of a sebaceous gland from a dog, showing heavy *Demodex* infestation © Eye of science, science photo library.

The exacerbation of acne in V15 aligns with research supporting a causal link between *Demodex* and acne (29–31). Viewed in cross-section, Figure 3 shows canine *Demodex* or “mange mites” tightly packed in a sebaceous gland. This also reminds us that acne treatments sometimes cause an initial exacerbation before improvement prevails, such as is seen with oral isotretinoin^11^. As isotretinoin is thought to reduce sebum production, the initial flare may denote a panic reaction by *Demodex* to the dwindling supply of nutrients (67). “Acneiform dermatitis”, a reported side effect of topical steroids^12^, may also be explained in terms of *Demodex* proliferation (26) due to local immunosuppression (33, 68–70) though signs may not be apparent while the medication is being used. The contraindication of immunosuppressants where infection is present^12^ might wisely be considered in cases where demodicosis has not been ruled out.

Caution therefore seems warranted when considering the Coston treatment for eye or respiratory symptoms in patients with significant skin disease and protocols for treating *Demodex*-related conditions should include measures for preventing or managing any initial exacerbation.

### 6.3 Clues for *Demodex* presence in the skin

Ectoparasites may be unpredictable due to the range of subtypes and their levels of reactivity. Melatonin increases mobility and reproduction in invertebrates (7), so nocturnal or early morning symptoms may be a feature. Skin symptoms include itching, burning, rash (39), scaling, dryness, lichenification (25), and acneiform outbreaks (31). Sequencing of mitochondrial DNA has shown *D. folliculorum* to be more metabolically active and likely to cause type I allergic reactions such as erythema and itching. By comparison, *D. brevis* focus more on producing enzymes for digesting chitin, host skin and serum molecules to facilitate penetration deep into the skin^13^ (20, 36). Therefore, while itching may be a strong clue to *Demodex* presence, absence of itching or negative skin surface tests should not exclude ectoparasites from a differential diagnosis. While cracking and fissure formation seem not to have been directly related to *Demodex* presence, lipid-loss and lichenification are likely to cause dryness and loss of elasticity, possibly leading to the cracking and flaking seen in eczema, ichthyosis and cheilitis.

Flares and periods of remission without identifiable reason, or where an environmental factor can be pinpointed, may be further signs of ectoparasite presence^14^. Fluctuations may be related to the tendency of *Demodex* to synchronise their life cycles (14). Coordinated shedding of allergens at regular intervals during moulting (9, 14) and mass rupture on death (16), are likely to amplify the allergenic impact.

Many parasites must migrate to specific sites or tissues within the host. Such “site specificity” is a common feature in many parasitic infections; for sexually reproducing parasites it facilitates finding a mate (58). Localisation of a skin condition is therefore another clue that ectoparasites may be present, and lesions with a well-defined map-like edge, as seen in the images of Volunteers V1 and V7^15^ may denote such colonisation^16^, as may changes in melanisation^17^ (71). It seems likely that a threat to survival would cause *Demodex* to move to another area, perhaps explaining the spread of symptoms seen with Volunteers V15 & V16. The potential benefit in making a timely and correct diagnosis cannot be overstated^18^.

Medicinal treatments for human demodicosis have been reviewed by Lam et al. (19, 72) and include both oral and topical preparations of metronidazole, ivermectin, and topical preparations of permethrin, lindane, benzyl benzoate and crotamiton. Treatment with oral ivermectin is given weekly and may be needed for six weeks or more, depending on *Demodex* density (73). This allows eggs and larvae, which are resistant to ivermectin, to mature and become susceptible to treatment.

### 6.4 *Demodex* & the immune system

*Demodex* mites have colonized mammalian hair follicles and sebaceous glands for millions of years; their presence being generally tolerated by the host immune system (74). The balance between asymptomatic colonization and pathogenic infestation is likely to be determined by both host and *Demodex* factors (35). Accounts of the complex interactions between *Demodex* and their host's immunological defence mechanisms are already available (26, 33, 35, 70, 72, 74–77). Comparison between human and canine demodicosis (35, 76) reveals a similar ability of *Demodex* to trigger and evade innate and adaptive immune responses in both humans and dogs (35), possibly even suppressing the innate host response to evade expulsion (70).

Inflammatory responses are triggered when *Demodex* cause mechanical damage by using their claws and piercing mouthparts to penetrate the dermis, destroying epithelial cells and ingesting the contents (11, 12, 72). Perifollicular inflammation may be the result of this process occurring inside hair follicles (13, 70). Extrafollicular mites and their debris, containing chitin (29), crystalline waste products, and microbes for which they may be vectors^19^ (78–80) can trigger the inflammation cascade via the toll-like receptor 2 (TLR2) innate immunity pathway (35, 72) or stimulate a granulomatous foreign body reaction. Proteins obtained from bacteria carried by *Demodex* have also been observed to trigger inflammatory reactions, causing neutrophils to migrate, degranulate and release cytokines (81).

Mechanical damage to the eye adnexa includes blockage of meibomian glands (72) increasing the risk of evaporative dry eye symptoms (82). Digestive proteases and lipases released by *Demodex**^20^*** may trigger host protease-activated receptors, cause anti-microbial peptides to be secreted and upregulate pro-inflammatory cytokines (72). In people with dry eye syndrome, lachrymal proteases are known to damage the ocular surface (82), warranting investigation for correlation with *Demodex* presence.

Keratinocytes and sebocytes both feature type 2 toll-like receptors which span the cell membrane. When they detect *Demodex* chitin it triggers an innate immune response (74, 77). This is thought to be the main mechanism for controlling mite population (74). Increased TLR2 production has been identified in papulopustular rosacea, which is strongly associated with *Demodex* presence (12); changes in production and distribution of TLR2 are also seen in atopic dermatitis, contact dermatitis and psoriasis (83), which are currently rarely ascribed to *Demodex*. However, *Demodex* may secrete bioactive molecules that affect TLR2 receptor expression (74) as a means of countering this phenomenon.

It has been shown that type 2 innate immunity reduces skin inflammation caused by *Demodex*. Decreased type 2 cytokine expression is observed in patients with *Demodex*-associated rhinophyma, affecting follicles of the nose, while activation of group 2 innate lymphoid cells (ILC2s), interleukin-13 (IL-13), and its receptor, IL-4Ra-IL-13Ra1, limit proliferation and spread of mites (75). Conversely, an absence of type 2 cytokines allows overgrowth of *Demodex,* with lymphocytic infiltrates, marked ILC2 activation and the development of inflammatory symptoms (75). Th2 cytokines have been implicated in asthma due to their role in the complex process of immunoglobulin E (IgE) production, and activation of mast cells and eosinophils (84).

The carbohydrate-like Tn antigen expressed by *Demodex* can modulate the secretion of pro-inflammatory mediators such as IL-8 and tumour necrosis factor (TNF)-*α* from the pilosebaceous unit of the host, which interferes with the innate immune response of the host to facilitate the invasion and population expansion of *Demodex* (29, 70). Acaricidal treatment decreases the antigenic load and reverses T-cell exhaustion, leading to a clinical cure (74).

An increased rate of lymphocyte apoptosis has been found to relate to *Demodex* density with the functional activity of leucocytes being significantly lower in infested individuals. This local immunosuppression attributed to *Demodex* may facilitate their survival in host skin. Secondary immunosuppression may also trigger demodicosis following corticosteroid use, cytostatic therapy or immunodeficiency disease (33). Analysis of the *Demodex* transcriptome has confirmed that nine “*Der f”* HDM allergens are highly expressed in *D. folliculorum,* and a link with erythema, papules, itching, and other symptoms of type I allergic reactions has already been proposed (36). However, the lack of a skin-prick test for *Demodex* allergy currently prevents screening for *Demodex* allergy and assessment of cross-reactivity between *Demodex* and HDM allergens.

The Retzingers' Acari Hypothesis III (66) proposed that, in atopic dermatitis and related conditions, the immune response is targeting infestation by “vector active acarians” and their dietary elements, thus stimulating production of IgE as part of the “*atopic march”*. This theory is developed in their Acari Hypothesis IV which proposes that mites and ticks are major causes of allergy through their “pathogenic payload” and that humans, monkeys and apes have evolved the eccrine system of sweat glands to deter infestation (85).

During infestation with *Demodex*, and in conditions where *Demodex* have been implicated, IgE is frequently elevated. IgE concentrations correlate with severity of atopic dermatitis (86) and are raised in patients with allergic asthma and rhinitis, who are allergic to HDMs (87). IgE has been found to be increased in patients with papulopustular rosacea compared with control groups in line with *Demodex* infestation (88) and in immunocompetent mice infested with *Demodex,* where significantly increased IgE concentrations fell after treatment with imidacloprid-moxidectin, a veterinary antiparasitic treatment (89).

High numbers of *Demodex* have been reported to induce proinflammatory cytokine secretion, whereas lower numbers did not (74). The phenomenon of life-cycle synchronicity (14) is likely to amplify any such reaction. In advanced disease in dogs, significantly elevated expression of TLR2, transforming growth factor (TGF)-β, and IL-10 and reduced expression of TLR6 have been found in the peripheral blood mononuclear cells (PBMCs) (90). It is thought that overexpression of the TLR2 gene might be responsible for *Demodex-*induced clinical manifestations, while down regulation of TLR4 and TLR6 gene expression and induction of systemic TGF-β and IL-10 could be strategies used by *Demodex* mites to avoid immune attack (34, 90). The phenomenon of T-cell exhaustion, which can be seen in advanced disease, features low IL-2 levels alongside high IL-10 and TGF-β production by lymphocytes, as described in other viral and parasitic diseases (16, 29, 74).

### 6.5 Safety and merits of the Coston treatment, using petroleum jelly

#### 6.5.1 Patient acceptability and potential effect on quality of life, in adults and children

The Coston Treatment is only applied at night, and the jelly is wiped or washed away on waking. All volunteers, including the children, readily accepted the treatment if the rationale was explained. Rapid relief of troublesome symptoms provided ongoing incentive. Full-scale clinical trials, which include a process for counting *Demodex*, would provide a more accurate assessment of safety, efficacy, and patient acceptability. The potential impact on patients' quality of life cannot be overstated. Even among our volunteers were cases where utter misery was alleviated. The impact on a child of having her mother bring an eye bag into school every day, or an academic not being able to work at a computer screen, the stigma and discomfort of skin conditions, and the impact and risks from respiratory inflammation, are clear too. If a cheap product which can be supplied without prescription can be shown to reduce ocular and respiratory inflammation, it could prove helpful in low-income countries where such treatments are most needed. Whether it would deter other eye-seeking parasites also merits investigation.

#### 6.5.2 Reduction in GP prescribing of allergy-related medicines and antibiotics

**Figure 4:**
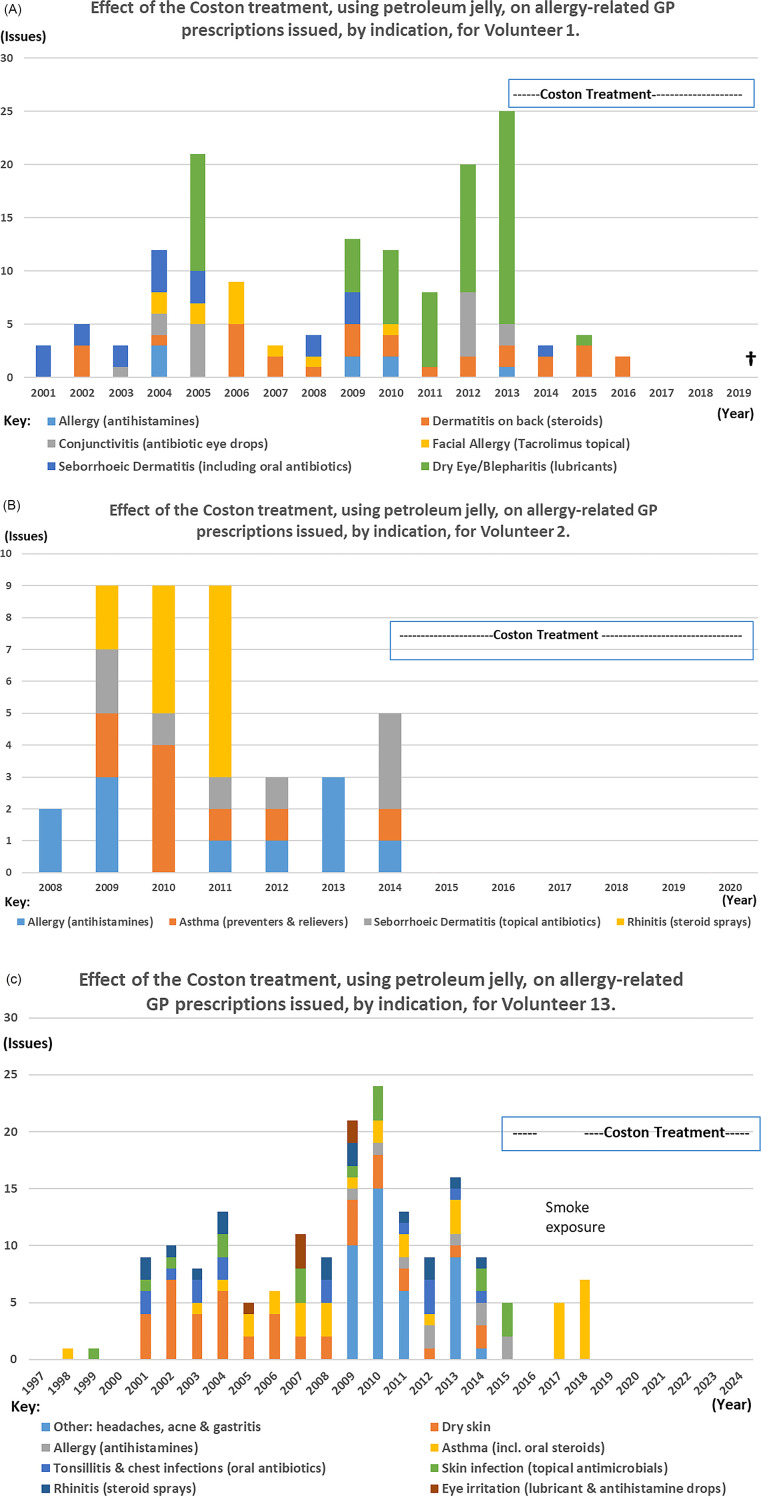
Graphs to show the impact of the Coston technique on the number of allergy-related items issued by the GP for Volunteers 1, 2 and 13 for the years for which data were available. Years of birth for V1, V2 and V13 were 1928, 2000 and 1997 respectively. **(A)** Effect of Coston treatment, using petroleum jelly, on allergy-related GP prescriptions issued, by indication, for Volunteer 1. **(B)** Effect of Coston treatment, using petroleum jelly, on allergy-related GP prescriptions issued, by indication, for Volunteer 2. **(C)** Effect of Coston treatment, using petroleum jelly, on allergy-related GP prescriptions issued, by indication, for Volunteer 13.

Subject to further assessment, the Coston technique, using petroleum jelly, is potentially a simple, cheap and effective first step in the treatment of dry eye and blepharitis. Volunteers 1–14 all reported needing fewer anti-allergy or dry eye treatments which provides a key economic incentive for further investigation. Retrospective analysis of prescribing data for Volunteers 1 and 2 and 13 is shown in Figure 4. These graphs portray almost total eradication of the need for ocular lubricants, antihistamines, nasal sprays and in V2 and V13 for asthma medication too; a phenomenon reinforced by anecdotal accounts from other volunteers. The role of *Demodex* as a vector for other micro-organisms (78) may explain why no further antibiotics were prescribed for these three volunteers, and merits further research.

Comparing the five-year periods immediately before and after starting continuous treatment, the mean number of anti-allergy and antimicrobial medicines prescribed per year for Volunteer 1, reduced from 15.6 (range 8–25) to 1.8 issues (range 0–4), representing an 88.5% decrease in prescribing. For Volunteers 2 and 13, mean issues fell from 5.8 (range 3–9) and 14.2 (range 9–24) respectively, to zero, representing 100% reductions in relevant prescribing for both people.

#### 6.5.3 Pharmaceutical considerations in the use of petroleum jelly

Petroleum jelly, a by-product of the oil industry, is a purified mixture of mineral oils and natural waxes. It has low allergenic potential and does not require preservatives due to the absence of water. As an inert vehicle for drug delivery in many ophthalmic preparations it comes into contact with the cornea and protects it (91). Available all over the world at minimal cost, it can be supplied without a prescription. Due to the physical action and complete lack of any active substance, it poses no risk of causing antimicrobial resistance and will not provide any sustenance for lipid-loving creatures such as *Demodex*. The cases described show that physical entrapment to prevent *Demodex* from mating could be a useful way to eradicate them from the eyelashes, allowing pharmacological agents to be reserved for more serious conditions. Like many cosmetic products applied externally to the eyelid margins of closed eyes, it does not need to be sterile though good handwashing before application is prudent. A patient advice sheet should be provided to explain the rationale for treatment, and how to apply it.

Despite the names 'soft paraffin', “petrolatum”, or “petroleum jelly”, the manufacturer's hazard assessment states that it is “non-flammable”^21^. All pharmaceutical emollients are required to carry a flammability warning, whether they contain paraffin derivatives, plant oils or animal fats, due to the reported accelerant action of emollients in general on the ignition and speed at which fabrics burn^22^. This warning appears not to be required for soft paraffin in its pure form, which is described as an “occlusive” rather than an “emollient”, so the packaging does not carry a flammability warning.

## 7 Suggestions for further research

Results from our volunteers, collected retrospectively and informally over several years, appear to reveal previously overlooked potential for *Demodex* to cause harm and suggest that their characteristics should be much more widely studied.

Trials on the safety and effectiveness of Coston's technique using petroleum jelly in blepharitis, dry eye syndrome, and other eye conditions linked to *Demodex* presence, seem warranted. Manipulation methods such as eyelash twirling (50) or lateral tension (51) may be the most accessible and acceptable techniques for counting mites, though PCR (92) or ELISA^23^ may also be feasible.

If the Coston technique can be shown to reduce *Demodex* count, a larger study should investigate possible wider clinical and economic benefits, including impact on prescription requests for medications for allergies, rhinitis, asthma and on quality of life. Other key outcomes may include reductions in surgery for nasal congestion and antibiotic use for skin and eye infections, with all the associated implications for microbial resistance. Whether a course of treatment before ocular surgery reduces post-operative inflammation or consequential dry eye symptoms, also merits investigation.

Alongside formal research, alerting clinicians to signs of ectoparasite activity may prove beneficial across a range of medical specialities. Closer links with arachnologists and acarologists, entomologists and veterinary ectoparasitologists would expedite our learning and help develop and normalise testing procedures. Use of the Coston petroleum jelly treatment as a deterrent for other eye-seeking parasites in low-income countries may also merit consideration.

Testing for ectoparasite presence and clinical trials of antiparasitic agents in a range of skin conditions would seem prudent. These should include atopic dermatitis, acneiform conditions, urticaria, angioedema, psoriasis, vitiligo, melanomas, and other conditions characterised by clear delineation with inflammation or pigmentation changes. Testing for *Demodex* 18s rRNA in recalcitrant sinusitis and in lung aspirates of patients with severe asthma or multi-resistant pneumonias could inform treatment, giving a positive impact on patient outcomes, reducing expenditure on medicines and hospital services, and help in the battle against antimicrobial resistance.

## 8 Falsifiers which could disprove the hypothesis

The hypotheses proposed would be falsified if the following research outcomes were established:

1. A suitably powered trial using a verified means of counting *Demodex* in eye lash follicles would find no significant reduction in *Demodex* count after a one-month course of petroleum jelly, correctly applied every night.
2. A longer-term trial in asthma and rhinitis patients would show no difference in symptoms or any reduction in use of symptomatic treatments between a group treated to reduce ocular *Demodex* and an untreated group.
3. *Demodex* proteins and debris would never show allergenic potential in a ’skin prick' test in atopic patients, if one should become available.
4. PCR testing for *Demodex* species in the nasal secretions of rhinitis patients, or in sputum samples from asthma and pneumonia patients, would always be negative.
5. Verified skin tests such as the standardised skin surface biopsy (93), PCR for *Demodex* 18s rRNA (65, 92), standard biopsy or deep skin scrape (94), or confocal microscopy (95) performed by fully trained individuals would find no evidence of *Demodex* in patients with dermatitis or acneiform conditions and symptoms would not respond to topical or oral antiparasitic treatments.

## 9 Limitations

This report is a retrospective account of unexpected clinical changes which occurred when a group of individuals, linked only by social contact with the author, used the treatment informally, in line with their individual preferences. Their demographics ranged widely in terms of age (9–84 years) and underlying health status. There was no access to specialist assessment of *Demodex* levels before or after treatment, which would be required in a clinical trial. With one exception, volunteers did not adhere to the advice to apply the petroleum jelly for 28 days. Some used shorter courses when required and some opted to use it long term. Formal trials would help to determine the most appropriate duration of treatment for specific indications and the likelihood of relapse.

## 10 Conclusions & summary

Applying petroleum jelly to the eyelash roots at night may reduce ocular *Demodex* levels by immobilising them when they roam at night to mate. This simple technique is based on Coston's observations in 1967 (10) that ointments in general were particularly effective if applied in this way. However, the technique is seldom used. The self-application by fascinated volunteers, accumulated over a 10-year period, has demonstrated potential effectiveness for dry eye disease and blepharitis, and therefore merits further investigation. Unexpected improvements in ocular allergies including angioedema, periocular and seborrhoeic dermatitis and acne were also seen, plus a striking resolution in rhinitis and asthma. Volunteers also described wider benefits to their wellbeing; a child no longer needed to use a heated “eye bag” in school, and an author returned to using her laptop and wearing contact lenses. Reduction in nocturnal coughing and a mother's perception of improved sleep quality is also described. The use of petroleum jelly in this way appeared acceptable to children and the elderly alike.

*Demodex* levels were not measured in these volunteers because their experiences were reported retrospectively, so a direct association between mite levels and symptom severity has not been established. However, the treatment had good clinical effect which the volunteers reported as correlating closely with changes in their compliance.

The unexpected outcomes in our 16 volunteers point to a mechanism whereby *Demodex* debris and allergenic proteins, including HDM-type “*Der f”* allergens, are transported via lachrymal fluid into nasal passages. Inhalation may then cause further inflammatory conditions including rhinitis and asthma. This is proposed as a hypothesis, and initial research methods to test and to falsify this hypothesis have been suggested. The reduction in GP prescribing for allergic and inflammatory conditions including dry eye, blepharitis, rhinitis and asthma, and of antimicrobials for eye and skin infections, if confirmed by further research, may provide an economical way to reduce prescribing and clinic costs. It may also help in ongoing campaigns to reduce antimicrobial use by physically removing a vector. Counselling to explain the technique and the rationale for treatment seems important, ideally supported by provision of a patient advice sheet.

The two cases where moderate acne and dermatitis flared and spread may demonstrate the phenomenon of parasites reacting adversely to an environmental threat to their survival. They also highlight the reported, but apparently not well-recognised, links between acne, dermatitis and *Demodex* (29–31, 65, 66). Clinical strategies to reduce the risk of a flare may need to be devised. Routine testing and increased clinical awareness of the signs of parasite presence may reduce the risk of dermatological or respiratory demodicosis being overlooked.

In addition to the already-established links between *Demodex* and several ocular and skin conditions, the unexpected benefits reported by the volunteers may also provide clues for a wider pathogenic role for *Demodex* and their debris in causing systemic inflammation. This may not be limited to the respiratory system because entry into the gastrointestinal tract seems equally likely. If these connections are confirmed, we may start to recognise a “*Demodex* Syndrome” where inflammatory conditions can be related to *Demodex* presence, particularly where an environmental factor, including variation in available nutrients, steroid hormones or immune response, can be identified.

Use of nightly petroleum jelly for dry eye and blepharitis has the advantage of being cheap, potentially very effective, and accessible without prescription, even in low-income countries without risk of causing resistance. It may also reduce use of steroids or other immunosuppressants which are generally contraindicated if an infestation or infection is suspected. The favourable risk-benefit ratio of this physical method may allow empirical treatment as the relationship between *Demodex* and ocular discomfort gains recognition. Further studies are required to confirm the impact of Coston's technique, using nightly petroleum jelly, on *Demodex* count and ocular symptoms. Wider potential benefits in allergic conditions including rhinitis and asthma, and other measures of health and wellbeing, such as GP prescribing rates and quality of life, remain to be assessed.

## Data Availability

The raw data supporting the conclusions of this article will be made available by the authors, without undue reservation.

## Footnotes

^1^See Supplementary S1 Figure 1A

^2^Please see Supplementary S1A for more information on digestive enzymes and pre-oral digestion.

^3^Please see Supplementary S1B for more information on vector status.

^4^Lowery RS. Ed. Law SK, Rapuano CJ, Dahl AA. Adult Blepharitis. *Medscape.* https://emedicine.medscape.com/article/1211763-overview Updated 17/8/23, accessed 3/1/25.

^5^Blepharitis: *NICE Clinical Knowledge Summary*. https://cks.nice.org.uk/blepharitis#!scenario. Updated September 2024, accessed 3/1/25.

^6^Please see Supplementary S2, figures 1A & 1B for images of Volunteer 1

^7^Geisse LJ. The Vaseline Routine. *Am. Ac. of Ophthalmol. Eye Net Magazine*. July 2023 (Letter). https://www.aao.org/eyenet/article/letters-28

^8^Busby TL. Anti-demodectic active agents and topical compositions for the treatment of demodicosis in humans and animals. (2016) US 20160287566 A1 (Patent application) https://patents.justia.com/patent/20160287566

^9^Please see Supplementary S1C on environmental reactivity for further information.

^10^For more information on veterinary parallels with human dermatoses please see Supplementary S1D

^11^Isotretinoin soft capsules 20mg (Roaccutane). *Summary of Product Characteristics*. Updated 31/10/23 accessed 4/1/25 https://www.medicines.org.uk/emc/product/6470/smpc#UNDESIRABLE_EFFECTS

^12^Mometasone 0.1% Ointment *Summary of Product Characteristics* https://www.medicines.org.uk/emc/product/9994/smpc#gref Updated 3/1/25, accessed 5/1/25

^13^Please see Supplementary S1A for more detail.

^14^Please see Supplementary S1C for more detail.

^15^Images may be found in Supplementary S2 Figures 1A, 3C),

^16^With thanks to Dr Miranda Whitten, Molecular Entomologist, Swansea University, for this information (personal communication December 2020).

^17^Please see Supplementary 1E on arthropods and melanin.

^18^For more information on diagnosis, and the possible impact of missing a diagnosis, please see Supplementary S1F & 1G

^19^For more detail on the vector status of *Demodex* please see Supplementary S1B.

^20^For more detail on enzymes and pre-oral digestion please see Supplementary S1A.

^21^Unilever Vaseline Petroleum Jelly Cosmetic Product Information Sheet. Updated 20/1/20, obtained from Unilever 14/7/23. Previous (2010) version available at https://safety365.sevron.co.uk/substances/accessSDS/SDS-20928-56a50fd4966712.91522705

^22^Emollients and risk of severe and fatal burns: new resources available - GOV.UK MHRA update 20 May 2021 to *Drug Safety Update* (2020) 14 (1): 6. Accessed 5/1/25.

^23^Lubna F. Diagnosis of canine demodicosis by ELISA. *(*2016 *Doctoral dissertation*, PVNR TVU). https://krishikosh.egranth.ac.in/items/2604ec3f-ad74-4d53-ba57-7d33bbbef3e0

## Author disclaimer

The views and ideas expressed in this article and any appendices are those of the author and not of her employer. All content presented is for informational and educational use only, and for the purpose of stimulating research; no part is intended or implied to be a substitute for professional medical advice, diagnosis, or treatment. It is assumed that healthcare professionals will use their clinical judgement, knowledge and expertise when deciding whether it is appropriate to use any part of this paper to influence their practice. The use of petroleum jelly as described in the text has not yet been subjected to formal assessment of safety and efficacy in clinical trials. Patients should always seek the advice of their physician or other qualified healthcare provider if they have questions or concerns and should not stop taking their current medication because of anything they have read in any part of this paper or any appendix to it. To the extent legally permissible, the author, people acknowledged, and Cardiff and Vale University Health Board disclaim any responsibility and any liability whatsoever for damages arising out of any clinical decisions based on use, inappropriate use or disregard of the information provided herein.
