# Supplementary material for: Reducing ocular Demodex using petroleum jelly may alleviate dry eye syndrome, blepharitis, facial dermatoses, ocular and respiratory allergies, and decrease associated prescribing: a hypothesis
Source: Front Allergy. 2025 Aug 20;6:1576102. doi: 10.3389/falgy.2025.1576102 (PMC12405285; doi:10.3389/falgy.2025.1576102)
Supplement: Supplementary file 1 [file Datasheet1.pdf]

Supplement 1 – extra information to assist understanding of Demodex behaviour:

A. Enzymes and pre-oral digestion
B. *Demodex* as vectors
C. Environmental reactivity & nutritional requirements
D. Demodicosis - veterinary parallels with human dermatoses
E. *Demodex* and melanin
F. Diagnosis of *Demodex* in the skin
G. Missed diagnoses, formication and zoonotic mite infestations

A. Enzymes and pre-oral digestion

As seen with spiders and many insects, *Demodex* digest their nutrients 'pre-orally' using a pharyngeal pump to emit digestive enzymes. These include 'immunoreactive' lipases to break down sebum (1) and proteases to acquire proteins for their chitin exoskeletons (2,3). *D. brevis* carry genes for proteases, which digest serum, chitin and host skin cells, facilitating skin penetration (4). Higher levels of matrix metalloproteases (MMPs) such as MMP-9 have been found in the tears of individuals with ocular *Demodex* compared to those who were *Demodex*-free (5). MMPs are cited as being one of many classes of proteases secreted into the tears in dry eye disease and loss of ocular surface barrier function appears to correlate with MMP levels. (6). Research to investigate the source of these MMPs would inform remaining debate about the role of *Demodex* in dry eye disease.

B. *Demodex* as vectors

Demodex act as mechanical vectors for a range of microbes possibly including *Streptococci* and S*taphylococci* (7,8) which are commonly implicated in microbial blepharitis (9,10). Microbes may adhere to the *Demodex* exoskeleton or be ingested (7) to serve as digestive aids, as is proposed with *Corynebacterium kroppenstedtii* (11,12). Other bacteria associated with *Demodex* include *Propionibacterium acnes, Corynebacterium spp.* (13) *Bacillus oleronius* (14), *B. pumilus, B. simplex, B. cereus* (12), *Wolbachia* (15), and *Helicobacter* (8). They have also been identified as vectors for the *SARS-CoV-2* COVID-19 virus (16) and other viruses in common with ticks, which are well-known vectors of disease (17).

C. Environmental reactivity and nutritional requirements

It is highly likely that *Demodex* will be very reactive to environmental changes. External factors determined by the host include use of soap and hygiene routines, exposure to sunlight and any other potential threats including antiparasitic agents. Internal factors include nutrient supply and hydration, but *Demodex* may also be affected by the availability of a plethora of other elements and compounds that they are likely to acquire from their hosts. These may include steroid hormones needed for moulting (18) and trace metals including iron, copper, zinc and cobalt required for diverse processes including enzyme production (19). Micro-invertebrates like *Demodex,* which only lay eggs once in

their life cycle, are more vulnerable to stressors than multiparous species (20), making them more likely to react to environmental changes. House dust mites may clump together if a stress-signal is triggered (21), though it is unclear whether *Demodex* share this survival response.

D. Demodicosis – veterinary parallels with human dermatoses

A role for *Demodex* in atopic dermatitis is now recognised (22,23). The clinical parallels with canine demodicosis are striking. Despite the different level of detail shown in Supp.1 Figs. 1 A-C, the similarity between human, canine and feline *Demodex* can be observed. They are usually typed according to the length of the abdomen, or 'opisthosoma', though polymorphism can occur. Whether the terminus is cone-shaped or finger-shaped may provide be a clearer indication of genotype (24).

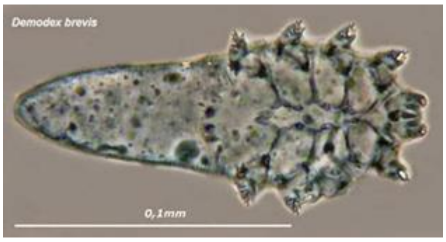

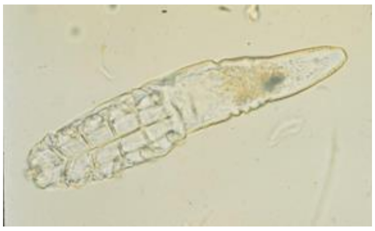

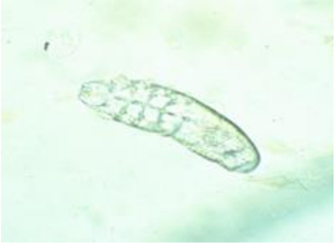

Supp.1 Fig 1A *Demodex brevis* (human)<br>Photograph by Austin Whittall (in public domain)	Supp.1 Fig 1B. *D canis* (canine)<br>© Dr Alice Jeromin [1]	Supp.1 Fig 1C. *D gatoi* (feline)<br>© Dr Alice Jeromin[1]

In humans and dogs, localised demodicosis occurs most commonly on the face, especially around the eyes and in the corners of the mouth (25,26). Further spread in dogs includes the trunk, legs, and ear canals, often with severe involvement of the paws. Common clinical findings in canine demodicosis include 'erythema, alopecia, macules and plaques, comedone formation, hyperpigmentation, lichenification and scarring' (27). Demodicosis of the paws may parallel skin conditions affecting the hands or feet in humans and merits consideration as part of the differential diagnosis.

Pustular demodicosis, or 'secondary pyoderma' in veterinary medicine, appears to have features aligning with severe acne and hidradenitis suppuritiva or 'acne inversa' in humans. Demodicosis is described as 'frequently including deep folliculitis, furunculosis with cellulitis, with regional lymphadenomegaly and fever' (27).

The preferred technique for diagnosing demodicosis in animals is the 'deep skin scrape', where the skin is squeezed firmly to express mites. Oil is then used to transfer scrapings from the scalpel to the microscope slide. Other tests include trichograms, test strips, and exudate collection for microscopy. In rare cases where these tests find no mites, skin biopsy may be required (28).

[1] With thanks to Dr Alice Jeromin, Veterinary Dermatologist, Ohio

E. *Demodex* and melanin

During infection, injury and the death process, most invertebrates produce melanin to protect themselves from bacterial and fungal infections and from UV radiation. Melanins are also used for hardening the exoskeleton, colour patterning, organogenesis and as an innate defence mechanism to immobilise and suffocate other microbes (29). Sequencing of the *Demodex* genome suggests that biochemical pathways for UV protection and synthesis of melanin have been lost (4,30), raising the prospect that *Demodex* may obtain melanin from their host. Conditions which feature changes in melanisation, such as psoriasis, vitiligo, melasma, and even malignant melanoma, might therefore be regarded with suspicion, particularly where a clear map-like delineation occurs.

F. Diagnosis of *Demodex* in the skin

In humans, the simplest techniques for diagnosis of *Demodex* in the skin include using a comedone extractor or the 'modified standardised skin surface biopsy'. This involves sequential layers of epidermis being removed for microscopy by applying cyanoacrylate glue spread on a microscope slide (31). Direct microscopic evaluation (32) relies on effective skin scraping, or epilation, to collect samples. Visualisation of living mites may be easier using dark field microscopy due to their bioluminescence (33). High-definition optical coherence tomography, *in vivo* confocal laser scanning microscopy (8), and polymerase chain reaction (PCR) testing for *Demodex* 18s rRNA (22,34) are higher cost options. Enzyme-linked immunosorbent assay (ELISA) to detect antibodies to *Demodex* antigens[2] may also be feasible. However, highly specific testing for *D. folliculorum* may risk not detecting other medically important ectoparasites acquired by zoonosis from other animals[3] or birds (35). The discrepancy between human medicine, where a positive result is classed as more than five mites per cm^2 (32), and veterinary medicine, where finding just one mite triggers treatment (28), begs reconsideration.

G. Missed diagnoses, formication and zoonotic mite infestations

With increased publicity on social media, many people with intractable skin inflammation are learning about *Demodex* and self-diagnosing the cause of their symptoms. As *Demodex* colonisation is so widespread, many will be correct in their reasoning. The current unavailability of reliable testing and the lack of confidence among clinicians to diagnose demodicosis based on symptoms and history, can

---

[2] Lubna F. Diagnosis of canine demodicosis by ELISA. (2016 *Doctoral dissertation*, PVNR TVU). https://krishikosh.egranth.ac.in/items/2604ec3f-ad74-4d53-ba57-7d33bbbef3e0

[3] Elverhaug V. *Demodex* in humans and their clinical implications: an overview of current research on various aspects on human *Demodex* infestations. *Master's Thesis*, University of South-Eastern Norway Faculty of Health and Social Sciences (2020). https://openarchive.usn.no/usn-xmlui/handle/11250/2725774

lead to frustration among patients and erosion of the doctor/patient relationship. Facial symptoms like those seen in our volunteers are likely to impact quality of life and mental health.

Patients may occasionally report an agonising feeling 'like ants crawling under the skin', perhaps without visible inflammatory changes. In these cases, non-specialist testing is likely to fail, leaving the clinician to conclude that the patient is deluded (36). Whether this 'formication' is purely physical or a subclinical immune response is not clear. However, it does have an established link with blepharitis (37). This connection must bring the safety of diagnoses such as 'delusional parasitosis' into question. Similar concerns could be raised about 'Matchbox Syndrome' (38), where desperate patients try to capture minute or microscopic creatures in a small container and present it at clinic as evidence of their infestation. Both diagnoses can affect patients physically and mentally and may lead to inappropriate initiation of antipsychotic medication. Use of reliable testing for ectoparasites or obtaining a thorough history, including potential exposure to bird or tree mites, as described by Coston in 1967 (39), may help to avoid this devastating scenario. This is an area which requires much more research, better testing and raised awareness among clinicians.

References

1. Jimenez-Acosta F, Planas L, Penneys N. *Demodex* mites contain immunoreactive lipase. *Arch Dermatol.* (1989) 125(10):1436–1437. doi:10.1001/archderm.1989.01670220134028
2. Nicholls SG, Oakley CL, Tan A, Vote BJ. *Demodex* species in human ocular disease: new clinicopathological aspects. *Int Ophthalmol.* (2017) 37: 303–312 doi: 10.1007/s10792-016-0249-9
3. Forton FMN. Papulopustular rosacea, skin immunity and *Demodex*: pityriasis folliculorum as a missing link. *JEADV.* (2012) 26: 19-28 doi: 10.1111/j.1468-3083.2011.04310.x
4. Hu L, Zhao Y, Niu D, Gong X, Yang R. *De novo* transcriptome sequencing and differential gene expression analysis of two parasitic human *Demodex* species. *Parasitol Res.* (2019) 118: 3223–3235 doi: 10.1007/s00436-019-06461-0
5. Zhang X-B, Ding Y-H, He W. The association between *Demodex* infestation and ocular surface manifestations in meibomian gland dysfunction. *Int J Ophthalmol*. (2018) 11: 589–592 doi: 10.18240/ijo.2018.04.08
6. Wolffsohn JW, Arita R, Chalmers R, Djalilian A, Dogru M, Dumbleton K et al.. TFOS DEWS II diagnostic methodology report. *Ocul Surf.* (2017) 15(3): 539-574. ISSN 1542-0124 doi: 10.1016/j.jtos.2017.05.001
7. English FP, Iwamoto T, Darrell RW, DeVoe AG. The vector potential of *Demodex folliculorum*. *Arch Ophthalmol.* (1970) 84(1):83–85 doi:10.1001/archopht.1970.00990040085020
8. Liang H, Randon M, Michee S, Tahiri R, Labbe A, Baudouin C. *In vivo* confocal microscopy evaluation of ocular and cutaneous alterations in patients with rosacea. *B J Ophth.* (2017) 101(3):268-274. UI: 27222243 doi:10.1136/bjophthalmol-2015-308110
9. Fromstein SR, Harthan JS, Patel J, Opitz DL. *Demodex* blepharitis: clinical perspectives. *Clin Optom.* (2018) 10:57–63 doi: 10.2147/OPTO.S142708
10. Liu J, Sheha H, Tseng S. Pathogenic role of *Demodex* mites in blepharitis. *Curr Opin Allergy Cl.* (2010) 10 (5): 505-510 doi: 10.1097/ACI.0b013e32833df9f4
11. Clanner-Engelshofen BM, French LE, Reinholz M. *Corynebacterium kroppenstedtii* subsp. *demodicis* is the endobacterium of *Demodex folliculorum*. *JEADV.* (2019) 34:1043-1049. doi: 10.1111/jdv.16069

12. Tatu AL, Nwabudike LC. Reply to: Kubiak K et al. Endosymbiosis and its significance in dermatology. *JEADV*. (2018) 32(9): e346-7. doi: 10.1111/jdv.14921.
13. Zhu M, Cheng C, Yi H, Lin L & Wu K. Quantitative analysis of the bacteria in blepharitis with *Demodex* infestation. *Front Microbiol*. (2018) 9: 1719. doi: 10.3389/fmicb.2018.01719
14. Lacey N, Delaney S, Kavanagh K, Powell FC. Mite-related bacterial antigens stimulate inflammatory cells in rosacea. *Brit J Dermatol.* (2007) 157 (3): 474-481. doi: 10.1111/j.1365-2133.2007.08028.x
15. Elston CA, Elston DM. *Demodex* mites. *Clin Dermatol*. (2014) 32 (6): 739-743. doi: 10.1016/j.clindermatol.2014.02.012
16. Tatu AL, Nadasdy T, Nwabudike LC. Chitin-lipid interactions and the potential relationship between *Demodex* and SARS-CoV-2. *Dermatol Ther*. (2021) 34(3): e14935. doi: 10.1111/dth.14935.
17. Neva FA, Brown HW. (1994) Basic Clinical Parasitology. 6th edn. Appleton & Lange, Norwalk CT. ISBN 0-8385-0624-0 p302.
18. Cohen E. Chapter 43 - Chitin. In: Resh VH, Cardé RT, editors. *Encyclopedia of Insects (Second Edition).* Cambridge MA: Academic Press. (2009) P. 156-157. ISBN 9780123741448. doi: 10.1016/B978-0-12-374144-8.00052-7
19. Khan S, Lang M. A comprehensive review on the roles of metals mediating insect-microbial pathogen interactions. *Metabolites*. (2023) 13(7):839. doi:10.3390/metabo13070839
20. Fajana HO, Gainer A, Jegede OO, Awuah KF, Princz JI, Owojori OJ, Siciliano SD. *Oppia nitens* C.L. Koch, 1836 (Acari: Oribatida): current status of its bionomics and relevance as a model invertebrate in soil ecotoxicology. *Environ Toxicol Chem.* (2019) 38:2593-2613. doi: 10.1002/etc.4574
21. Skelton AC, Cameron MM, Pickett JA, Birkett MA. Identification of neryl formate as the airborne aggregation pheromone for the American house dust mite and the European house dust mite; (Acar: Epidermoptidae). *J Med Entomol.* (2010) 47(5):798-804. doi:10.1093/jmedent/47.5.798
22. Edslev SM, Andersen PS, Agner T, Saunte DML, Ingham AC, Johannesen TB, Clausen M-L. Identification of cutaneous fungi and mites in adult atopic dermatitis: analysis by targeted 18S rRNA amplicon sequencing. *BMC Microbiol.* (2021) 21: 72. doi: 10.1186/s12866-021-02139-9
23. Retzinger AC, Retzinger GS. The Acari Hypothesis, III: atopic dermatitis. *Pathogens.* (2022) 11(10):1083. doi: 10.3390/pathogens11101083
24. Zhao YE, Hu L, Ma JX. Molecular identification of four phenotypes of human *Demodex* mites (Acari: Demodicidae) based on mitochondrial 16S rDNA. *Parasitol Res*. (2013) 112: 3703-11. doi: 10.1007/s00436-013-3558-1
25. Chen W, Plewig G. Human demodicosis: revisit and a proposed classification. *B J Dermatol*. (2014) 170(6), 1219–1225. doi:10.1111/bjd.12850#
26. Rufli T, Mumcuoglu Y. The hair follicle mites *Demodex folliculorum* and *Demodex brevis*: biology and medical importance. *Dermatology.* (1981) 162(1):1-1. doi:10.1159/000250228
27. Foley R, Kelly P, Gatault S, Powell F. *Demodex:* a skin resident in man and his best friend. *JEADV*. (2021) 35(1):62-72. doi: 10.1111/jdv.16461
28. Mueller RS, Rosenkrantz W, Bensignor E, Karas-Tezcza J, Paterson T, Shipstone MA. Diagnosis and treatment of demodicosis in dogs and cats. Clinical consensus guidelines of the World Association for Veterinary Dermatology. *Vet Dermatol*. (2020) 31: 4–e2. doi: 10.1111/vde.12806
29. Whitten MMA, Coates CJ. Re-evaluation of insect melanogenesis research: views from the dark side. *Pigm Cell Melanoma R*. (2017) 30:386–401. doi: 10.1111/pcmr.12590
30. Smith G, Manzano-Marín A, Reyes-Prieto M, Sofia Antunes CSR, Ashworth V, Goselle ON et al. Human follicular mites: ectoparasites becoming symbionts. *Mol Biol Evol*. (2022) 39:6.msac125. doi: 10.1093/molbev/msac125

31. Lacey N, Russell-Hallinan A, Powell, FC. Study of *Demodex* mites: challenges and solutions. *JEADV*. (2016) 30(5):764-775. doi: 10.1111/jdv.13517
32. Rychlik K, Sternicka J, Zabłotna M, Nowicki RJ, Bieniaszewski L, Purzycka-Bohdan D. The prevalence of *Demodex* spp. infestation in dermatological patients in northern Poland. *Life*. (2024) 14(9):1196. doi: 10.3390/life14091196
33. Aytekin S, Yaşar S, Göktay F, Güneş P. Spontaneous fluorescence of *Demodex* in the dark. (letter) *JEADV*. (2016) 30: 320–38. doi:10.1111/jdv.12776
34. Kasetsuwan N, Kositphipat K, Busayarat M, Threekhan P, Preativatanyou K, Phumee A, Siriyasatien P. Prevalence of ocular demodicosis among patients at Tertiary Care Center, Bangkok, Thailand. *Int J Ophthalmol.* (2017) 10(1):122-127. doi:10.18240/ijo.2017.01.20
35. Boseret G, Losson B, Mainil JG, Thiry E, Saegerman C. Zoonoses in pet birds: review and perspectives. *Vet Res* (2013) 44:36. doi: 10.1186/1297-9716-44-36
36. Wong J, Koo J. Delusions of parasitosis. *Indian J Dermatol*. (2013) 58(1):49-52. doi: 10.4103/0019-5154.105309
37. Mastrota KM. Blepharitis diagnosis may lead to formication. *Optom Times*. (2014) 6(6):17. https://search.proquest.com/docview/1545685839?accountid=15585
38. Freudenmann RW, Kölle M, Schönfeldt-Lecuona C, Dieckmann S, Harth W, Lepping P. Delusional parasitosis and the matchbox sign revisited: the international perspective. *Acta derm-venereol.* (2010) 90(5):517-9. doi:10.2340/00015555-0909
39. Coston TO. *Demodex folliculorum* blepharitis. *Trans Am Ophth Soc*. (1967) 65: 362-392. https://www.ncbi.nlm.nih.gov/pmc/articles/PMC1310279/pdf/taos00034-0371.pdf
