# Supplementary material for: Reducing ocular Demodex using petroleum jelly may alleviate dry eye syndrome, blepharitis, facial dermatoses, ocular and respiratory allergies, and decrease associated prescribing: a hypothesis
Source: Front Allergy. 2025 Aug 20;6:1576102. doi: 10.3389/falgy.2025.1576102 (PMC12405285; doi:10.3389/falgy.2025.1576102)
Supplement: Supplementary file 2 [file Datasheet2.pdf]

Supplement (S)2: Case reports - full accounts of use of Coston treatment with petroleum jelly by volunteers with dry eye syndrome, blepharitis, rhinitis, allergies, acne and asthma.

Volunteer 1: Blepharitis and dermatitis in an 84-year-old lady.

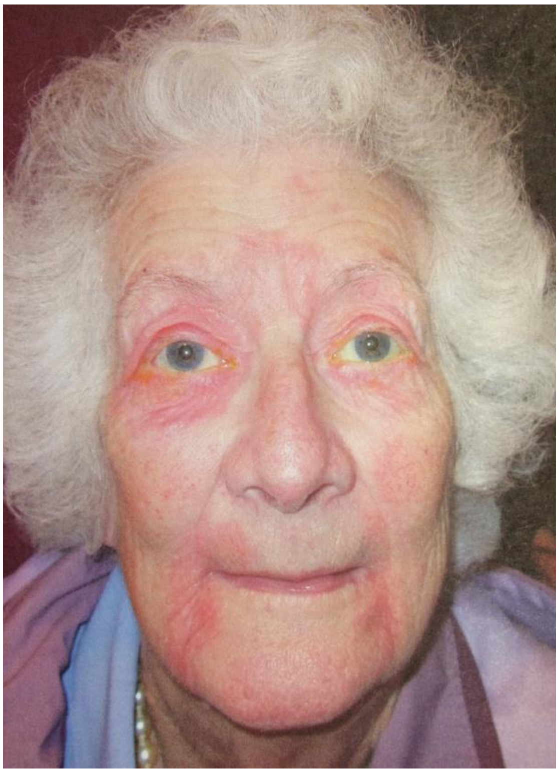

Figure 1(A). Volunteer 1, at age 76 in 2004, with chronic blepharitis, perioral dermatitis and seborrhoeic dermatitis, prior to starting 8 years of treatment with an immunosuppressant ointment. ©D. Senior-Fletcher. All rights reserved.

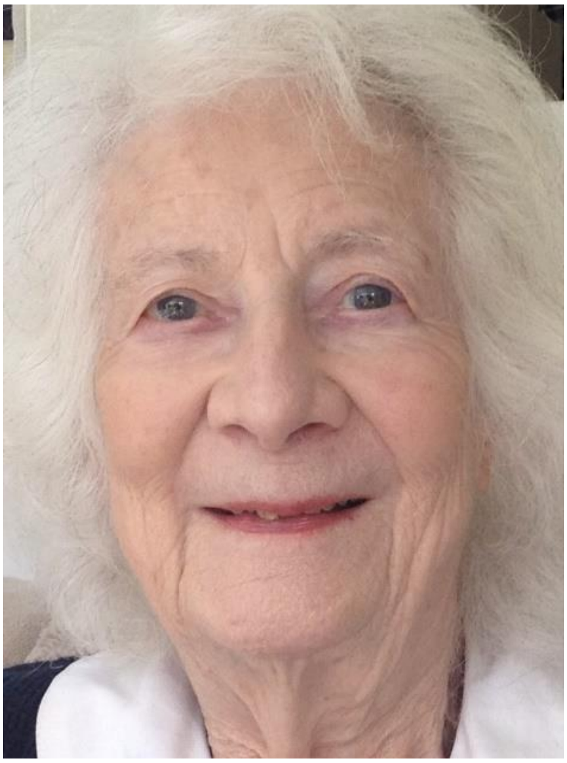

Figure 1(B). Volunteer 1 at aged 90 in 2018 after 6 years of Coston's treatment using nightly petroleum jelly as monotherapy. © D. Senior-Fletcher. All rights reserved.

The first volunteer, as shown in Figure 1(A), had suffered for three decades with dry eyes, blepharitis, and severely itching patches of seborrhoeic dermatitis around her eyes, ears, and mouth. Her medical history included thyrotoxicosis, chronic gastric ulceration with perforation, frequent migraines, arthritis, and hypertension. In her early 70s she grew increasingly forgetful and, in 2008 at age 80, she was diagnosed with mixed Alzheimer's Disease and vascular dementia. Early cataracts were identified in 2012 at age 84. Over a period spanning 30 years she had been prescribed multiple courses of topical and oral antibiotics, antihistamines, topical antifungals, and steroids, to treat her eye and skin conditions. She was taking regular medication for gastric ulcers, hypertension, osteoporosis, and thyroid hormone replacement. This severe condition was treated with tacrolimus ointment by her General Practitioner (GP) which provided some relief for eight years before the Coston treatment was started in late 2013, supervised, to ensure concordance.

V1's blepharitis and dermatitis improved dramatically within a few days. After a month, the frequency was reduced to every three nights, arbitrarily, to prevent reoccurrence, and continued for the rest of her life. Retrospective analysis of her GP records confirmed that she required ocular antibiotics six times and ocular lubricants twelve times in 2012. In 2013 ocular antibiotics were issued twice and ocular lubricants twenty times but with few further issues after 24/9/13. During the next six years, she was prescribed only one further pack of an ocular lubricant which remained unused. No antibiotics, topical or oral, for her eye or skin conditions, or further antihistamines were requested from her GP. Her general health also improved, allowing the proton pump inhibitors for her gastritis to be stopped; her migraines resolved, and arthritis in her hands improved too. Unlike her half-sister and a cousin, her dementia did not deteriorate beyond short term memory loss. She remained able to live alone, with support, maintaining her ability to answer some crossword clues and catch a ball reliably while seated. In 2019, her optometrist confirmed that her cataracts, first diagnosed in 2012, had not developed and that her vision was clear. She died in November 2019, aged 91, following a hip fracture, intra-operative stroke and pneumonia.

Volunteer 2: Blepharitis, nasal congestion, and asthma in a 13-year-old boy

This teenager had a 4-year history of blepharitis with itching, inflamed eyes, and debris in the lashes, treated unsuccessfully with eyelid-cleansing solution. He also suffered rhinitis and asthma, requiring regular corticosteroid nasal sprays, oral antihistamines, and inhaled salbutamol. Hypertrophy of the nasal mucosa, considered severe enough for surgery, was diagnosed; skin prick tests confirmed allergy to cats, grasses, and house dust mites.

V2 started the Coston treatment with petroleum jelly in late 2013 or early 2014 at the age of 13. Remarkably, in addition to his eye symptoms resolving, his nasal obstruction also cleared fully within 3-4 days. Retrospective review of his GP notes confirmed the observation that he no longer required nasal sprays after starting the Coston treatment. Some years later he remarked that he no longer had asthma. One repeat issue of antihistamines and one of salbutamol in 2014 were precautionary and not used. V2 continued to use the petroleum jelly treatment every night into adulthood, and reports that it prevents return of his allergy symptoms.

Volunteer 3: Nasal congestion, seborrhoeic dermatitis, & acne in a 58-year-old male

This healthcare worker tested Coston's treatment in 2017 for his chronic rhinitis, suffered since the age of 14. He reported with surprise that his concurrent facial acne and seborrheic dermatitis had resolved quickly, and that his nasal congestion cleared shortly afterwards. He admitted erratic compliance, finding it *"effective if used for a few days, just now and then".* A year later he re-treated himself for relapsed nasal congestion, and stated "*Just like the last time, after one night of treatment I woke with a clear head and a clear nose. It seems hard to believe that it could work so fast."* He has since recommended this treatment for blepharitis in his immunocompromised patients. In 2024 he reported verbally that he had not used the Coston treatment for several years, and had developed mixed psoriasis and seborrhoeic dermatitis, requiring treatment with topical immunosuppressants.

Volunteer 4. Seborrhoeic dermatitis in a 50-year-old male

This healthcare worker started using the treatment in about 2018 for his nasal congestion. He reported that application for 2-3 weeks had resolved both the nasal congestion and his seborrhoeic dermatitis, and that he only needed to reapply it occasionally. In 2024 he reported not needing to use the treatment but was prepared to do so if his symptoms returned.

Volunteer 5. Acne and dry eye syndrome in a 16-year-old female

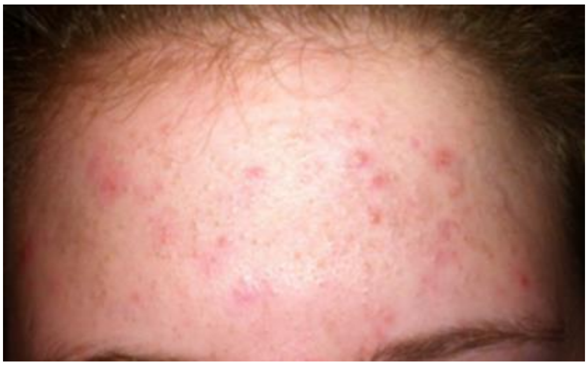

Figure 2(A). Forehead of Volunteer 5 showing the severity of her chronic acne before starting the Coston treatment. (©Image courtesy of V5. All rights reserved.)

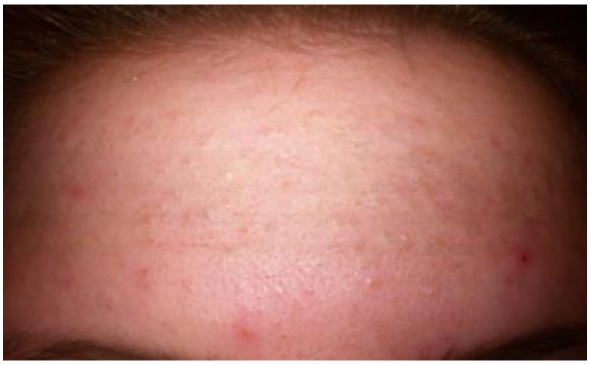

Figure 2(B). Forehead of Volunteer 5, showing improvement in acne after one week of the Coston treatment. (©Image courtesy of V5. All rights reserved.)

This teenager, with moderate chronic facial acne, used petroleum jelly on her lashes and directly on acne on her face and upper body. Due to the rapid success of this treatment in clearing her skin (Figure 2B), supported by resurgence when she briefly stopped treatment, she taught her school friends to do the same, and declared that this simple treatment had "*transformed [her] life*". Her optometrist coincidentally reported resolution of her dry eye syndrome, which had not been mentioned previously. Two years later, after continued use, she reported a new outbreak of acne on her back. This was disappointing but indicates that *Demodex*, if indeed they were the cause of these changes, may, like other parasites, migrate to a new area on the host, to improve their chance of survival.

Volunteer 6: Angioedema in a young woman

In 2015, this 23-year-old healthcare worker with a history of eczema and hay fever suffered severe swelling of both eyes. She required emergency treatment with corticosteroids in a hospital A&E department where she was started on high dose fexofenadine. Subsequently she was diagnosed with idiopathic angioedema by an immunologist. During the next two years she made two failed attempts at stopping the antihistamines, finding that symptoms returned after one day. In 2017 she used the Coston treatment for 28 days then withdrew the antihistamine successfully over several days. On follow-up three years later, she reported having suffered no further attacks of angioedema and had not needed to repeat the treatment.

Volunteer 7: Ocular, peri-ocular, and peri-oral erythema in a young woman

The next volunteer, also a 23-year-old healthcare worker, had a history of mild eczema since childhood. She had suffered four allergic reactions affecting the face and eyes, since the age of 19 (Figures 3(A) and 3(B). In 2014, severe conjunctivitis was treated unsuccessfully with topical chloramphenicol and steroids, then successfully with sodium cromoglicate.

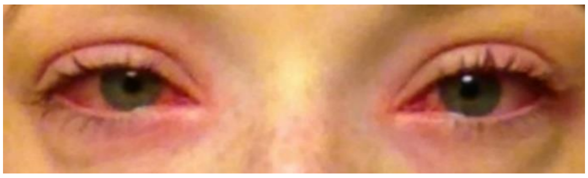

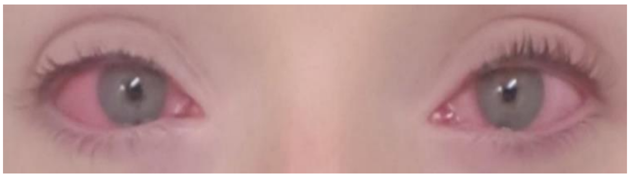

Figure 3(A). Volunteer 7 diagnosed with allergic conjunctivitis. (©Photographs courtesy of Volunteer 7, all rights reserved.)	Figure 3(B). Volunteer 7 with scleritis-type reaction. (©Photograph courtesy of Volunteer 7, all rights reserved.)

Two years later V7 developed severe itching and erythema around her eyes, ears, and mouth with a well-defined map-like edge (Figure 3(C)). Potent topical steroids, antifungal creams and tacrolimus ointment were ineffective, so oral prednisolone was required. No specific allergens could be identified.

During a subsequent flare, three months later, V7 started using the Coston treatment, approximately three times a week which she continued with good effect for over a year (Figure 3(D)). Without further application, she remained symptom-free for two years before another flare around the eyes required treatment with oral steroids. Subsequently, she re-commenced the Coston treatment and continues to use it intermittently whenever a flare is developing. She reports that it has reduced the severity of her flares (Figure 3(E)), which usually subside within a few days of restarting the treatment. Eczema on her arms has also resolved after using heavy ointments at night, in a similar approach to the Coston treatment.

Continued/

V7 Continued/

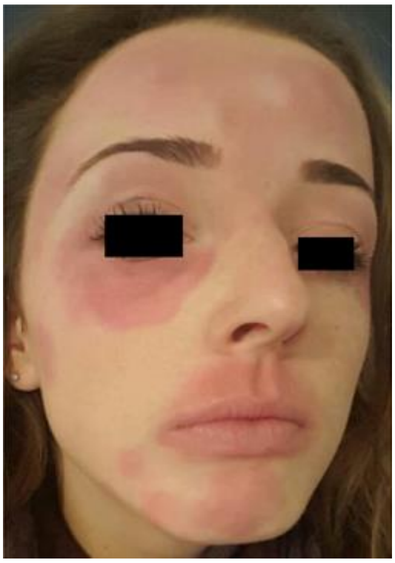

Figure 3(C). Volunteer 7 showing perioral and peri -ocular map-like erythema, with further spread to forehead and chin, clearly showing map-like distribution. (©Photograph courtesy of Volunteer 7, all rights reserved.)

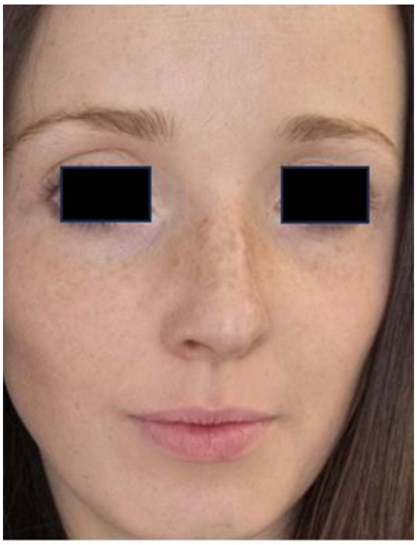

Figure 3(D). Volunteer 7 showing resolution of facial allergy with intermittent use of nightly petroleum jelly to prevent flares. (©Photograph courtesy of Volunteer 7, all rights reserved.)

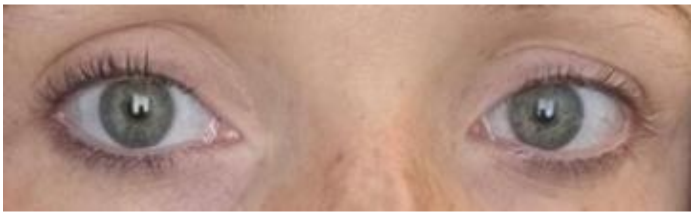

Figure 3(E). Resolution of symptoms in Volunteer 7 following treatment with petroleum jelly and ongoing intermittent use as prophylaxis. (©Photograph courtesy of Volunteer 7, all rights reserved.)

Volunteer 8. Severe blepharitis and asthma in a 9-year-old girl

From the age of three, V8 had suffered severe eye irritation with visible lash dandruff, and asthma-like nocturnal coughing, requiring multiple visits to her GP, and had an allergy to horses. Her mother noticed a link between eye symptoms and sugar intake. At age 6, an ophthalmologist diagnosed blepharitis and explained a connection with eczema and psoriasis, prescribing steroid eye drops and ointment, for use in severe outbreaks, and subsequently offering a 3-month course of antibiotics.

Instead, V8's mother tried eyelid hygiene and oral antihistamines without benefit, then bought an electrically heated eye mask. She supervised her daughter's use of it for 40 minutes a day, even at school during lunch breaks, but again, with limited benefit. Inhaled steroids and salbutamol were continued during this period for nocturnal cough and exercise-induced asthma.

Her mother reported that, within one week of starting the Coston treatment, V8 showed dramatic improvement in eye symptoms. After three months she was clear of styes, her nocturnal cough had abated, and she was sleeping more soundly. She grew *"several inches*" over the next 4-6 months, which her mother related to deeper sleep. Her mother also noticed that V8's blepharitis flared whenever treatment lapsed and that it resolved within three days of restarting supervised treatment

Without any use of the heated eye mask or antihistamines, V8 reportedly stayed clear of blepharitis and styes for over a year. She did not require salbutamol or inhaled corticosteroids, could play energetic team sports, and go horse riding without needing antihistamines. Her ophthalmologist confirmed complete resolution of her blepharitis.

Volunteer 9. Blepharitis, styes, skin irritation, ear inflammation, rhinitis, and asthma in an 11-year-old boy

This child, older brother of V8, had a similar history, with mild blepharitis and styes for two years, featuring significant discomfort and itching, plus nasal congestion. He suffered itching round his ears, which were "*frequently blocked*", and he required inhaled steroids and salbutamol for asthma. Again, after "*a few days*" of the Coston treatment, eye symptoms, nasal congestion and peri-auricular itching had eased. Longer term, he also stopped getting styes and his snoring resolved. Like his sister, he had grown approximately four inches in height since starting the treatment. His mother observed his symptoms and snoring return when she didn't monitor application, and that control returned within 3 days of re-starting supervision. She reported that he no longer uses any regular asthma medication except as a precaution if he catches a cold.

Volunteer 10. Ocular inflammation, asthma, and other allergic symptoms in a peri-menopausal woman

This non-healthcare worker, in her late 40s and mother of V8 & V9, described waking frequently with inflamed eyes, "*looking like a puffer fish*". Her history included earache, poor sleep, night sweats, anxiety, boils, and full body skin itching with '*blotches*', which she related to her time on hormone replacement therapy. She was also recovering from severe COVID-19 requiring hospitalisation four months earlier. She had suffered asthma from age 18 and had been treated in hospital for shortness of breath and hives, again triggered by allergy to horses. After 8-9 days of the Coston treatment with petroleum jelly, V10 claimed that her chest had cleared; the itching, boils and blotches had resolved, and that she was sleeping much more soundly. After three weeks she noticed her asthma had improved and she stopped needing her reliever medication. She reported her skin symptoms resolving, with no further itching at night. Eighteen months later, she was still using the treatment and remained untroubled by previous complaints, being particularly pleased that her eye inflammation had not recurred. She also reported no longer using inhaled steroids, except as a precaution during a cold or chest infection.

Volunteer 11. Dry eye syndrome in a perimenopausal woman

This academic and author with multiple allergies had a two-year history of severe dry eye syndrome, which prevented use of contact lenses and made screen work very uncomfortable. She suffered significant eye inflammation on waking, frequent episodes of conjunctivitis and recurrent eye infections. She had used antihistamines, ocular lubricants, sodium cromoglicate, and had increased her fluid intake, all without benefit. In 2014 she started the Coston treatment and reported reduced discomfort and inflammation, even after the first application. Within one week her dry eye symptoms had resolved fully, allowing her to wear her contact lenses again. She reported repeating the treatment whenever symptoms returned and that despite her initial low expectation of success, it had made *"a big difference"* to her. In 2024 she reported not having used further medication for dry eye syndrome, allergies or hay fever; she had not contracted another eye infection since starting the Coston treatment and was able to wear her contact lenses for much longer periods without discomfort.

Volunteer 12. Keratitis in a 52-year-old female with rheumatoid arthritis (RA)

This healthcare worker had a long history of ocular inflammation, starting with anterior uveitis at age 6. Her parents were told this signified a likelihood of RA in later life. In adulthood, she suffered uveitis and dendritic corneal ulcers at stressful times, treated with topical steroids, topical antibiotics, and antivirals. After diagnosis of RA at age 50, she was told she was losing her sight due to viral keratitis, despite having used oral antivirals and steroid eye drops long term. Her eyes appeared dry and irritated, so, in 2017, she started using the Coston treatment. She has since been told by her ophthalmologist that the keratitis has resolved and that her eyesight is no longer in danger. She continues oral antivirals and steroid drops as prescribed but also applies petroleum jelly every night, finding it prevents return of her dry eyes and ocular irritation. She also reports an apparent correlation between use of the treatment and reduced joint stiffness and pain related to her RA.

Volunteer 13. Asthma in a young woman

This arts graduate had a history of rhinitis, gastritis, and headaches from the age of 10, apparently coincident with developing acne on her forehead, and had suffered asthma-like symptoms since infancy. In 2015 she used the Coston treatment and successfully resolved her rhinitis and asthma, prior to starting university. However, treatment lapsed during her second year 2016-7, while studying abroad and sharing accommodation with cigarette smokers. Her asthma flared, requiring emergency courses of oral prednisolone, in addition to regular high dose inhaled corticosteroids, a long-acting beta agonist, and a leukotriene antagonist. Back in the UK on an unrecorded date in late 2018 or early 2019, she intensified use of the Coston treatment. She then withdrew her asthma and rhinitis medications slowly and completely over a period of a few weeks without further relapse. While prescribing data are incomplete due to living abroad again from 2019-22, she confirmed she had no further need for asthma treatments during that period.

Volunteer 14. Dry eye syndrome with excess eye watering in an adult female

This domestic cleaner and dog-walker had a long history of itching, watery, visibly sore eyes aggravated by cosmetics, plus severe seasonal hay fever featuring sneezing and rhinorrhoea. Within a few days of starting the Coston treatment in 2022, her eye symptoms greatly improved with no further itching or inflammation. In 2024 she reported that ongoing treatment kept her symptom-free and that she had not experienced any hay fever symptoms that year, to her great surprise.

Cases with adverse outcomes

Volunteer 15. Acne in a young woman

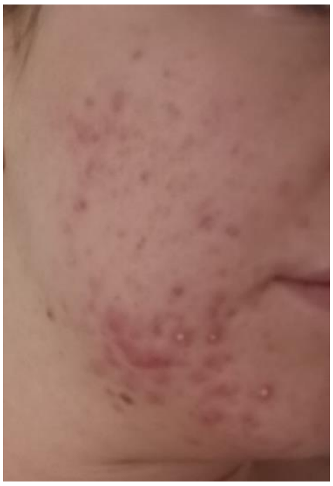

This data officer with a long history of severe acne was living away from home. On this occasion, the contact was indirect, and no explanation of the mechanism was given. About a week after starting the Coston treatment, she reported a severe flare of acne (Figure 4) featuring a deep crack at the corner of her mouth. She stopped treatment and recovered gradually without intervention.

Figure 4. Exacerbation of acne in Volunteer 15, following use of the Coston treatment with petroleum jelly, taken some time after the peak of the flare. (©Image courtesy of Volunteer 15. All rights reserved.)

Volunteer 16. Dry itching eyes and eczema in a 60-year-old diabetic

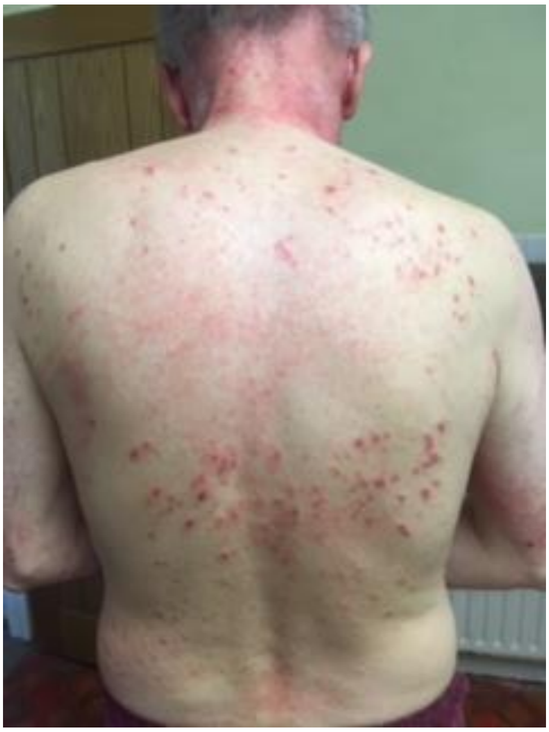

This scientist with type 1 diabetes, had a history of chronic eczema of the face, scalp and body, with dry, itching eyes. He reported little initial benefit from regular use of the Coston treatment. Subsequently, he increased the area treated, to include the full face and scalp, which resulted in a severe full-body flare (Figure 5), with intense itching and raised excoriated patches, even on untreated areas. A dermatologist performed a gentle, and therefore probably ineffective, skin scrape, but no *Demodex* were found. He was prescribed oral steroids and a potent topical steroid ointment to regain control.

Figure 5. Full body flare of eczema in Volunteer 16, following wider use of petroleum jelly on areas of pre-existing dermatitis on the face and scalp. ©Image courtesy of Volunteer 16, all rights reserved.

End of document
